# Computational prediction and validation of C/D, H/ACA and Eh_U3 snoRNAs of *Entamoeba histolytica*

**DOI:** 10.1186/1471-2164-13-390

**Published:** 2012-08-14

**Authors:** Devinder Kaur, Abhishek Kumar Gupta, Vandana Kumari, Rahul Sharma, Alok Bhattacharya, Sudha Bhattacharya

**Affiliations:** 1 School of Environmental Sciences, Jawaharlal Nehru University, New Delhi, 110067, India; 2 School of Life Sciences, Jawaharlal Nehru University, New Delhi, 110067, India

**Keywords:** U3 snoRNA, Guide/ orphan snoRNAs, *Entamoeba histolytica*

## Abstract

**Background:**

Small nucleolar RNAs are a highly conserved group of small RNAs found in eukaryotic cells. Genes encoding these RNAs are diversely located throughout the genome. They are functionally conserved, performing post transcriptional modification (methylation and pseudouridylation) of rRNA and other nuclear RNAs. They belong to two major categories: the C/D box and H/ACA box containing snoRNAs. U3 snoRNA is an exceptional member of C/D box snoRNAs and is involved in early processing of pre-rRNA. An antisense sequence is present in each snoRNA which guides the modification or processing of target RNA. However, some snoRNAs lack this sequence and often they are called orphan snoRNAs.

**Results:**

We have searched snoRNAs of *Entamoeba histolytica* from the genome sequence using computational programmes (snoscan and snoSeeker) and we obtained 99 snoRNAs (C/D and H/ACA box snoRNAs) along with 5 copies of Eh_U3 snoRNAs. These are located diversely in the genome, mostly in intergenic regions, while some are found in ORFs of protein coding genes, intron and UTRs. The computationally predicted snoRNAs were validated by RT-PCR and northern blotting. The expected sizes were in agreement with the observed sizes for all C/D box snoRNAs tested, while for some of the H/ACA box there was indication of processing to generate shorter products.

**Conclusion:**

Our results showed the presence of snoRNAs in *E. histolytica*, an early branching eukaryote, and the structural features of *E. histolytica* snoRNAs were well conserved when compared with yeast and human snoRNAs. This study will help in understanding the evolution of these conserved RNAs in diverse phylogenetic groups.

## Background

Small nucleolar RNAs (snoRNAs) are a special class of small non coding RNAs localized to the nucleolus. They belong to two major categories; box C/D and box H/ACA snoRNAs, based on the presence of short consensus sequence motifs [1]. H/ACA box snoRNAs guide the pseudouridylation while C/D box snoRNAs guide the site specific 2'-o-ribose methylation during post transcriptional modification of pre rRNA [2-4]. Such modification is accomplished by complementary base pairing between specific regions of the snoRNA and target RNA by the small nucleolar ribonucleoprotein complex which guides the modification of target RNA. Some snoRNAs are also known to perform functions other than the modification of ribosomal RNAs, e.g. U3, U17, U8, U14, and U22. The U3 snoRNA is an exceptional member of the box C/D class, and is involved in early pre rRNA cleavage in the 5’ external transcribed spacer (ETS) in yeast cells [5], mouse extracts [6], and Xenopus oocyte extracts [7]. Depletion of this snoRNA impairs the formation of mature 18 S rRNA [3]. Other exceptions include C/D snoRNA U8 [8], U22 [9] and an H/ACA snoRNA U17/snR30 [10] which are required for pre-rRNA cleavage. They are not involved in rRNA and nuclear RNA modification. Some snoRNAs are involved in both pre-rRNA cleavage as well as modification e.g. U14 (C/D) [11] and snR10 (H/ACA) [12]. Several snoRNAs lack any known target site, and are called orphan snoRNAs. These snoRNAs might have undiscovered functions, which may or may not concern rRNAs. Evidence in this respect is the role of orphan C/D box snoRNA (SNORD115) in regulation of alternative splicing [13].

Structural motifs are one of the important distinguishing features of snoRNAs. The characteristic structural motifs in C/D box snoRNAs are RUGAUGA for C box and CUGA for D box. In H/ACA box snoRNAs the H box is ANANNA and ACA box is ACA, arranged in a hairpin, hinge, hairpin, tail structure [14,15]. C/D box snoRNAs are about 60–100 bases in size, while H/ACA snoRNAs are 120–160 bases. Vertebrate snoRNAs are typically encoded from introns of protein coding genes [16] while in plants they are transcribed as polycistronic transcripts [17]. In yeast most of them are transcribed from independent promoters [18]. Amongst protozoan parasites, snoRNAs have been extensively studied in *Trypanosoma brucei*[19] and *Plasmodium falciparum*[20-22]. In the latter it was shown for the first time that snoRNA genes may be located in UTRs. Strikingly, both organisms showed a much larger number of methylation sites compared with pseudouridylation sites.

A number of bioinformatic tools are available for the scanning of genomic sequences for snoRNAs. These include Snoscan [23] and snoSeeker (CDSeeker and ACASeeker) [24] for the search of C/D and H/ACA box snoRNAs. In this study, we have carried out a genome wide analysis of the early branching parasitic protist *Entamoeba histolytica* for identification of C/D and H/ACA box snoRNAs in this organism. A computational search for structural motifs gave hits out of which false positives having no identifiable target sites were removed. This was achieved by aligning the rRNA of *E. histolytica* with rRNAs of five eukaryotic organisms *Arabidopsis thaliana, Caenorhabditis elegans, Drosophila melanogaster, Saccharomyces cerevisiae and Homo sapiens* separately, whose snoRNAs and target sites are already known [25-27]. The computational analysis was combined with experimental validation.

## Results and discussion

### Computational identification of putative snoRNAs from *E. histolytica* by snoscan and snoSeeker

**Figure 1:**
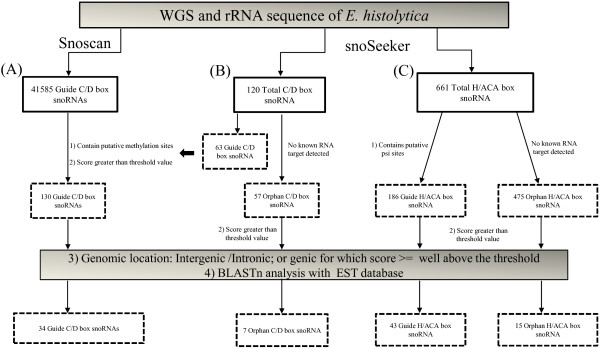
**Flowchart showing analysis with Snoscan and snoSeeker.** (**A**) C/D box guide snoRNAs predicted by Snoscan and final selection of candidate snoRNAs on the basis of indicated filters. (**B**) The initial count and the selected orphan C/D box snoRNAs using CDSeeker. (**C**) Initial count and final selection of H/ACA box snoRNAs using ACASeeker.

Target site modifications by snoRNAs are commonly conserved amongst distant eukaryotes [28]. We therefore selected five eukaryotic organisms: *A. thaliana, C. elegans, D. melanogaster, S. cerevisiae, H. sapiens*, whose methylation sites and pseudouridylation (psi) sites are known and used these to find putative sites in *E. histolytica* rRNA by aligning its 5.8 S, 28 S and 18 S rRNA sequences with rRNAs of the selected organisms separately (Additional file 1: Figure S1). Each of the mapped methylation and psi sites were picked as putative modification sites in *E. histolytica*. We could identify a total of 173 putative methylation sites and 126 putative psi sites in *E. histolytica.* A large fraction of these (53%) matched with yeast and human sites. 24 novel methylation sites were also found in *E. histolytica*. The programs snoscan and snoSeeker (CDSeeker); and snoSeeker (ACASeeker) were used to identify the putative sequences for C/D and H/ACA box snoRNAs respectively in *E. histolytica* whole genome. The initially predicted snoRNAs (41705 C/D box and 661 H/ACA box) were further analyzed to eliminate false positive candidates using the following criteria (Figure 1). Firstly, we selected snoRNAs that could target the putative modification sites obtained by aligning the rRNA of *E. histolytica* with the five organisms listed above. SnoRNAs that could potentially target 23 predicted methyl sites and 41 psi sites in *E. histolytica* were thus selected. Secondly, we set a threshold value, the final logarithmic odd score, that incorporated information from each of the snoRNA features and fetched out the snoRNAs having final score equal or more than the threshold value [24,26]. The threshold values used are given in “Methods”. Thirdly; we looked for the genomic localization of these snoRNAs and selected those coming from intergenic regions and introns. We also selected snoRNAs from genic regions for which the logarithmic odd score was well above the threshold (45 bits for H/ACA and 20 bits for C/D box snoRNAs) [24,26]. Lastly, we did BLASTn analysis of predicted snoRNAs with EST database of *E. histolytica*. All those snoRNAs giving hits with ESTs were discarded. Finally we obtained a total of 99 snoRNAs of which 41 were C/D box (34 guide and 7 orphan snoRNAs) and 58 were H/ACA box (43 guide and 15 orphan snoRNAs). We have named the genes encoding the putative snoRNAs so as to indicate firstly the type of snoRNA (Me or ACA), followed by species name (Eh) and the modification site in rRNA (where predicted) or orphan (where it is not known), e.g. ACA-Eh-SSU-1315 represents H/ACA type of snoRNA of *E. histolytica* which is predicted to modify SSU rRNA at position 1315 (Tables 1, 2, 3).

**Table 1:** **Box C/D snoRNA genes in *E. histolytica***

**snoRNA genes**	**Len.**	**Seq.**	**Modification**	**Antisense element**	**Scaffold**	**Start**	**End**	**Location**
	**(nt)**	**(%)**						
**Me-Eh-SSU-G1296	78	92%	SSU-G1296	12nt(5') 100%	DS571223	24176	24254	IR
			SSU-G1298	10nt(5’) 100%				
			SSU-G1195	10nt(5’) 100%				
Me-Eh-SSU-U1024	80	96%	SSU-U1024	14nt(5') 95%	DS571261	44605	44684	IR ●
			SSU-U1822	11nt(5’) 98%				
**Me-Eh-SSU-A83	78	100%	SSU-A83	16nt(5') 100%	DS571196	58225	58327	IR
			SSU-U87	12nt(5’) 100%				
Me-Eh-SSU-G41	68	93%	SSU-G41	11nt(5’) 100%	DS571147	177417	177350	IR
Me-Eh-SSU-A431	68	94%	SSU-A431	13nt(5') 100%	DS571331	10236	10303	IR
Me-Eh-SSU-U871	80	96%	SSU-U871	20nt(5’) 95%	DS571673	2402	2481	NA
*Me-Eh-SSU-G1535	82	93%	SSU-G1535	12nt(5') 100%	DS571215	31121	31040	IR
			LSU-G2053	9nt(5') 100%				
Me-Eh-SSU-A27	66	100%	SSU-A27	11nt(5') 100%	DS571226	26372	26307	IR
Me-Eh-SSU-A1830	83	88%	SSU-A1830	11nt(5') 100%	DS571152	29351	29433	EHI_049420 (+)
Me-Eh-SSU-A836	103	--	SSU-A836	13nt(5’)	DS571152	99242	99140	IR
Me-Eh-SSU-G1152	60	91%	SSU-G1152	12nt(3') 100%	DS571335	19522	19581	IR
Me-Eh-SSU-G628	97	--	SSU-G628	10nt(5’)	DS571451	15436	15532	IR
					DS571177	52928	52831	
Me-Eh-SSU-A1183	82	--	SSU-A1183	10nt(5’)	DS571164	22795	22876	IR
			SSU-G1836	13nt(5’)				
			SSU-A1485	9nt(5’)				
			LSU-A520	12nt(3’)				
			LSU-U1210	12nt(5’)				
			LSU-A145	10nt(3’)				
Me-Eh-SSU-A790	68	94%	SSU-A790	10nt(5’) 100%	DS571171	51701	51634	IR
			SSU-A1496	11nt(5’) 100%				
			LSU-A801	11nt(5’) 100%				
			LSU-A1834	10nt(5’) 100%				
			LSU-A2555	11nt(5’) 100%				
Me-Eh-SSU-C1805	63	96%	SSU-C1805	10nt(5') 100%	DS571145	496851	496789	IR
Me-Eh-LSU-A928a	69	97%	LSU-A928	11nt(5') 100%	DS571323	13072	13140	IR
Me-Eh-LSU-A928b	66	98%	LSU-A782	11nt(5') 100%	DS571163	50734	50669	IR
			LSU-A928	9nt(5’) 100%				
			LSU-A1034	9nt(5’) 100%				
Me-Eh-LSU-U1868	101	92%	LSU-U1868	13nt(5’) 92.3%	DS571175	28933	28833	IR
Me-Eh-LSU-U3580a	103	--	LSU-U3580	19nt(5’)	DS571304	677	575	IR
Me-Eh-LSU-U3580b	105	--	LSU-U3580	19nt(5’)	DS571305	36390	36494	IR
Me-Eh-LSU-A785	62	96%	LSU-A785	13nt(5') 100%	DS571416	15678	15739	IR
Me-Eh-LSU-G2958	70	97%	LSU-G2958	13nt(5') 100%	DS571205	22350	22419	IR
*Me-Eh-LSU-A3089	71	92%	LSU-A3089	11nt(5’) 100%	DS571180	41005	40935	IR
*Me-Eh-LSU-C2414	69	97%	LSU-C2414	11nt(5') 100%	DS571473	957	1025	IR ●
Me-Eh-LSU-G926	59	98%	LSU-G926	13nt(3') 100%	DS571150	13447	13389	IR
Me-Eh-LSU-U1018	69	--	LSU-U1018	11nt(5’)	DS571215	62034	62102	IR
			LSU-U2783	14nt(5’)	DS571316	3067	2999	
Me-Eh-LSU-G1028	61	87%	LSU-G1028	14nt(3’) 100%	DS571174	92482	92422	IR
Me-Eh-LSU-U1176a	109	94%	LSU-U1176	14nt(5') 100%	DS571307	17712	17820	IR ▲
Me-Eh-LSU-U1176b	109	94%	LSU-U1176	14nt(5') 100%	DS571419	10643	10535	IR
Me-Eh-LSU-U1176c	109	93%	LSU-U1176	14nt(5') 100%	DS571792	3710	3820	IR
Me-Eh-LSU-A2333	128	93%	LSU-A2333	12nt(3') 100%	DS571208	15564	15691	IR ●
**Me-Eh-LSU-A228	72	97%	LSU-A228	13nt(5') 100%	DS571397	17920	17991	EHI_003940
								Intron of gene
								40 S ribosomal protein S4, putative
**Me-Eh-5.8 S-U84	62	86%	5.8 S-U84	18nt(3’) 91%	DS571194	27534	27595	3’UTR
*Me-Eh-5.8 S-A92	115	85%	5.8 S-A92	11nt(5’) 94%	DS571180	76405	76291	EHI_118830 (−) ■

** snoRNAs validated by RT-PCR and Northern, * validated only by RT-PCR.

**Note:** “Len.” denotes length of the snoRNA genes, “Seq.” is sequence identity of corresponding snoRNA genes in *E. dispar*, “Antisense element” denotes length of antisense element in *E. histolytica* and its sequence identity with *E. dispar*. “IR”, intergenic region, “NA”, no annotation. snoRNA located close to ribosomal protein genes ●, downstream to rRNA methyltransferase gene ▲, close to C/D box snoRNP (fibrillarin) ■. (+) and (−) represents snoRNA in sense and antisense orientation with respect to host gene.

**Table 2:** **Box H/ACA snoRNA genes in *E. histolytica***

**snoRNA genes**	**Len**	**Seq**	**Modification**	**Antisense element**	**Scaffold**	**Start**	**End**	**Location**
	**(nt)**	**(%)**						
**ACA-Eh-SSU1315	121	96%	SSU1315	6 + 7nt (5’) 100%	DS571149	98793	98673	IR
ACA-Eh-SSU631	137	-	SSU631	6 + 5nt (3’)	DS572405	485	349	NA
		-	SSU1114	8 + 9nt (5’)	DS572405	485	349	
ACA-Eh-SSU1727	135	87%	SSU1727	9 + 5nt (5’) 93%	DS571346	12499	12633	IR/5'UTR
**ACA-Eh-SSU626	127	94%	SSU626	6 + 6nt(3') 100%	DS571463	13091	12965	IR
ACA-Eh-SSU461	142	-	SSU461	7 + 5nt (3’)	DS571171	90117	90258	IR
ACA-Eh-SSU1675	127	92%	SSU1675	5 + 9nt (3') 93%	DS571182	71521	71647	IR
*ACA-Eh-SSU526	126	94%	SSU526	7 + 5nt (5') 100%	DS571463	12972	13097	IR
*ACA-Eh-LSU3008	129	92%	LSU3008	7 + 5nt (5') 100%	DS571272	39423	39295	IR
ACA-Eh-LSU1172a	142	-	LSU1172	5 + 4nt (5’)	DS571149	73439	73580	IR
ACA-Eh-LSU1172b	141	-	LSU1172	5 + 4nt (3’)	DS571307	22719	22859	IR
ACA-Eh-LSU1107b	155	-	LSU1107	11 + 3nt (5’)	DS571159	2240	2086	IR
		-	LSU1172	6 + 8nt (3’)	DS571159	2240	2086	
		-	5.8 S52	8 + 3nt (3’)	DS571159	2240	2086	
ACA-Eh-LSU1650	118	89%	LSU1650	8 + 5nt (5’) 100%	DS571267	21025	21142	IR
ACA-Eh-LSU3087	129	92%	LSU3087	6 + 4nt (5') 100%	DS571178	75373	75501	IR
ACA-EH-LSU2791	161		LSU2791	6 + 7nt (5’)	DS571159	59530	59690	IR
ACA-Eh-LSU3155	151	88%	LSU3155	5 + 6nt (3') 91%	DS571255	1114	963	IR
ACA-Eh-LSU3221	152	79%	LSU3221	9 + 4nt (5’) 91.6	DS571339	14712	14561	IR
ACA-Eh-LSU1159a	154	-	LSU1159	4 + 5nt (5’)	DS571589	7973	8126	IR
					DS571660	2209	2056	
ACA-Eh-LSU2700	144	86%	LSU2700	8 + 3nt (3') 100%	DS571160	113417	113560	IR
	144		LSU1159	6 + 4nt (3') 100%	DS571160	113417	113560	
ACA-Eh-LSU1080	123	-	LSU1080	3 + 7nt (5’)	DS571228	4519	4641	IR
**ACA-Eh-LSU1343	137	-	LSU1343	5 + 5nt (5’)	DS571219	12011	11875	IR
ACA-Eh-LSU2997b	129	96%	LSU2997	5 + 4nt (5') 100%	DS571145	384477	384605	IR
ACA-Eh-LSU339	148	-	LSU339	5 + 4nt (5’)	DS571174	50939	50792	IR
ACA-Eh-LSU1123	148	-	LSU1123	4 + 7nt (5’)	DS571225	51991	52138	IR
ACA-Eh-LSU1005	148	-	LSU1005	4 + 5nt (3’)	DS571402	1263	1116	IR
ACA-Eh-LSU1236a	141	-	LSU1236	3 + 6nt (3’)	DS571481	789	649	IR
ACA-Eh-LSU1236b	141	-	LSU1236	3 + 6nt (3’)	DS571159	21643	21503	IR
ACA-Eh-LSU1107a	154	-	LSU1107	11 + 4nt (3’)	DS571208	46788	46941	IR/ 5'UTR
		-	SSU1114	8 + 9nt (5’)	DS571208	46788	46941	
**ACA-Eh-LSU2288	126	92%	LSU2288	4 + 9nt(5') 100%	DS571148	172182	172057	IR
			SSU1431	6 + 4nt (3') 90.0%	DS571148	172182	172057	
ACA-Eh-LSU1159b	153	-	LSU1159	5 + 5nt (5’)	DS572251	153	1	NA
		-	LSU3221	4 + 6nt (3’)	DS572251	153	1	
		-	SSU826	4 + 6nt (5’)	DS572251	153	1	
ACA-Eh-LSU2997a	122	-	LSU2997	5 + 6nt (5’)	DS572347	1128	1007	NA
					DS572347	800	679	
					DS572347	464	343	
					DS572347	132	11	
ACA-Eh-5.8 S80a	140	-	5.8 S80	5 + 9nt (5’)	DS571346	5092	4953	IR
ACA-Eh-5.8 S80b	132	-	5.8S80	5 + 6nt (5’)	DS571206	1568	1437	IR
		-	LSU3221	5 + 5nt (5’)	DS571206	1568	1437	
ACA-Eh-SSU740	141	92%	SSU740	4 + 7nt (3') 91%	DS571156	54460	54320	EHI_182810 (+)
*ACA-Eh-SSU188	135	93%	SSU188	6 + 3nt (5’) 89%	DS571501	5129	5263	EHI_172000 (+)
*ACA-Eh-SSU1216	142	77%	SSU1216	5 + 4nt (3’) 89%	DS571247	8141	8000	EHI_016340 (−)
ACA-Eh-SSU299	169	94%	SSU299	4 + 6nt (3') 100%	DS571161	119527	119695	EHI_142230 (+)
ACA-Eh-SSU1212	129	93%	SSU1212	9 + 7nt (3') 100%	DS571169	105772	105900	EHI_098580 (−)
**ACA-Eh-LSU2809	156	82%	LSU2809	12 + 3nt(3') 86.7%	DS571148	116513	116668	EHI_012330 (−)
ACA-Eh-LSU2335	131	93%	LSU2335	3 + 6nt (5’) 100%	DS571304	17766	17896	EHI_161910 (−)
ACA-Eh-LSU2493	135	87%	LSU2493	8 + 3nt (5’) 82%	DS571228	40854	40720	EHI_161000 (−) ●
ACA-Eh-LSU1176	157	97%	LSU1176	5 + 4nt (5') 100%	DS571185	32437	32593	EHI_104450 (+)
ACA-Eh-LSU2268	135	97%	LSU2268	7 + 3nt (3') 90%	DS571154	24191	24057	EHI_178500 (−)
*ACA-Eh-5.8 S84	152	82%	5.8 S84	7 + 5nt (5’) 92%	DS571169	105495	105646	EHI_098580 (−)

** snoRNAs validated by RT-PCR and Northern, * validated only by RT-PCR.

**Note:** “Len.” denotes length of the snoRNA genes, “Seq.” is sequence identity of corresponding snoRNA genes in *E. dispar*, “Antisense element” denotes length of antisense element in *E. histolytica* and its sequence identity with *E. dispar*. “IR”, intergenic region, “NA”, no annotation. snoRNA located close to ribosomal protein genes ●. (+) and (−) represents snoRNA in sense and antisense orientation with respect to host gene.

**Table 3:** **Orphan snoRNA genes (C/D and H/ACA) in *E. histolytica***

**snoRNA genes**	**Len**	**Seq**	**Modification**	**Antisense element**	**Scaffold**	**Start**	**End**	**Homology Yeast Human**	**Location**
	**(nt)**	**(%)**							
EhCDOrph1	95	95%	unknown	unknown	DS571162	42554	42648	unknown	EHI_155390 (+)
EhCDOrph2	87	94%	unknown	unknown	DS571301	21222	21308	unknown	IR
EhCDOrph3	107	94%	unknown	unknown	DS571358	4592	4698	unknown	IR
EhCDOrph4	91	96%	unknown	unknown	DS571422	5594	5684	unknown	IR
EhCDOrph5	84	94%	unknown	unknown	DS571468	9619	9702	unknown	IR
EhCDOrph6	94	--	unknown	unknown	DS571178	12358	12451	unknown	3'UTR/IR
EhCDOrph7	94	--	unknown	unknown	DS571178	13726	13819	unknown	3'UTR/IR
EhACAOrph1	115	91%	unknown	unknown	DS571172	5407	5293	unknown	IR
EhACAOrph2	135	93%	unknown	unknown	DS571155	108854	108988	unknown	IR/5’UTR ●
**EhACAOrph3	137	94%	unknown	unknown	DS571258	10028	9892	unknown	IR
EhACAOrph4	122	90%	unknown	unknown	DS571205	43143	43022	unknown	IR
EhACAOrph5	129	-	unknown	unknown	DS571332	15845	15717	unknown	IR
**EhACAOrph6	158	-	unknown	unknown	DS571298	19208	19365	unknown	IR
EhACAOrph7	130	-	unknown	unknown	DS571219	6608	6737	unknown	IR
EhACAOrph8	131	88%	unknown	unknown	DS571162	44597	44467	unknown	IR ●
EhACAOrph9	120	89%	unknown	unknown	DS571164	102500	102619	unknown	IR ●
EhACAOrph10	149	87%	unknown	unknown	DS571179	6844	6696	unknown	EHI_093690 (−) ●
EhACAOrph11	139	94%	unknown	unknown	DS571299	12352	12214	unknown	EHI_099700 (−)
EhACAOrph12	134	91%	unknown	unknown	DS571402	6404	6271	unknown	EHI_067510 (−) ●
**EhACAOrph13	137	95%	unknown	unknown	DS571501	3747	3883	unknown	EHI_171990 (+)
**EhACAOrph14	153	97%	unknown	unknown	DS571295	13935	14087	unknown	EHI_082520 (−)
*EhACAOrph15	148	91%	unknown	unknown	DS571166	95075	95222	unknown	EHI_127390 (−)

** snoRNAs validated by RT-PCR and Northern, * validated only by RT-PCR.

**Note:** “Len.” denotes length of the snoRNA genes, “Seq.” is sequence identity of corresponding snoRNA genes in *E. dispar*, “IR”, intergenic region, “NA”, no annotation. snoRNA located close to ribosomal protein genes ●. (+) and (−) represents snoRNA in sense and antisense orientation with respect to host gene.

We compared the predicted *E. histolytica* snoRNAs with those of *S. cerevisiae*[29], *H. sapiens*[30] and the two protozoan parasites (*T. brucei* and *P. falciparum*) on the basis of homology with conserved antisense sequences that guide the respective modifications for the two snoRNA classes (Table 4). We found 9 C/D guide snoRNAs out of 34 which showed homology with *P. falciparum* snoRNAs, and 10/34 which showed homology with *T. brucei* snoRNAs, while in yeast and human this number was 14/34 (with yeast) and 11/34 (with human). Only 4 *E. histolytica* H/ACA box snoRNAs out of 43 showed homology with *P. falciparum* snoRNAs and 2/43 showed homology with *T. brucei* snoRNAs, while the homology with yeast was 14/43 and with human was 18/43. The conservation of modification sites between these organisms was as follows. Of the sites predicted to be modified in *E. histolytica* rRNAs (47 methylation sites and 41 pseudouridylation sites), 16 methylation sites and 21 pseudouridylation sites were conserved in at least one of the other four organisms (Table 4). Taking the two modification sites together, 30 sites were conserved between *E. histolytica* and *S. cerevisiae*, 31 between *E. histolytica* and *H. sapiens*, 13 sites between *E. histolytica* and *P. falciparum,* and 12 sites were conserved between *E. histolytica* and *T. brucei.* Seven modification sites of *E. histolytica* were shared by all the four organisms. We also found 7 and 15 orphan snoRNAs in the C/D and H/ACA categories respectively. Orphan snoRNAs are important as they may act on RNA substrates other than mature rRNAs. As mentioned before, one of the roles of orphan snoRNAs is reported for human HBII-52 snoRNA [13], which is a C/D orphan snoRNA and regulates alternative splicing of the serotonin receptor 2 C. Similarly, some orphan H/ACA box snoRNAs may function in other aspects of RNA biogenesis. For example, the human U17 box H/ACA snoRNA and its yeast orthologue, snR30, plays an essential role in the nucleolytic processing of 18 S rRNA from pre rRNA. We checked for sequence complementarity of the antisense elements in our predicted orphan snoRNAs with the *E. histolytica* data base. For two C/D orphan snoRNAs (Additional file 2: Figure S2) the possible antisense element (upstream to D' box and/or D box) showed complementary base paring with mRNAs of EHI_192630 and EHI_008070 genes in *E. histolytica*. Further we checked whether the predicted orphan snoRNAs were found in the small RNA data base of *E. histolytica* (generated in our lab by next generation sequencing). We found that 14 of 22 orphan snoRNAs were detected in this data base.

**Table 4:** **Homology of *E. histolytica *snoRNAs and modification sites with selected organisms**

**snoRNA genes of *E. histolytica***	**Modification**	**Homology**				**Conservation of sites**
		**Yeast**	**Human**	***P. falciparum***	***T. brucei***	
Me-Eh-SSU-G1296	SSU-G1296	snR40	U232A	-	TB9Cs3C1	YHT
		18SG1271	18SG 1328		SSU Gm1676	
Me-Eh-SSU-A431	SSU-A431	snR87	U16	PFS11	-	YHP
		18SA 436	18SA 484	18S Am442		
Me-Eh-SSU-G1535	SSU-G1535	snR56	U25	snoR25	TB9Cs2C4	YHPT
		18SG 1428	18SG	G1674SSU	SSU Gm1895	
Me-Eh-SSU-A27	SSU-A27	snR74	U27	PFS4	TB8Cs2C1	YHPT
		18SA 28	27	18S Am28	SSU Am56	
Me-Eh-SSU-G1152	SSU-G1152	snR41	-	-	-	Y
		18SG 1126				
Me-Eh-SSU-A790	SSU-A790	snR53	-	-	-	Y
		18SA 796				
Me-Eh-SSU-C1805	SSU-C1805	snR70	U43	-	TB10Cs4C3	YHT
		18SC 1639	18SC 1703		SSU Um2123	
Me-Eh-LSU-A928a	LSU-A928	snR39	U32A	-	TB11Cs4C2	YHT
		28SA 807	28SA 1511		LSU5 Am1091	
Me-Eh-LSU-A785	LSU-A785	U18	U18A	PFS13	TB10Cs2C2	YHPT
		28SA 649	28SA 1313	28S Am728	LSU Am910	
Me-Eh-LSU-G2958	LSU-G2958	snR38	snR38A	PFS7	TB11Cs1C2	YHPT
		28SG 2815	28SG 4362	28S Gm3176	LSU3Gm1207	
Me-Eh-LSU-A3089	LSU-A3089	snR71	U29	PFS2	-	YHP
		28SA 2946	28SA 4493	18S A1129,28SAm3307		
Me-Eh-LSU-C2414	LSU-C2414	snR64	U74	PFS15, PFS16	TB10Cs1C1	YHPT
		28SC 2337	28SC 3820	28S Cm2632	LSU3 Cm538	
Me-Eh-LSU-G926	LSU-G926	snR39b	snR39B	PFS8	TB9Cs2C3	YHPT
		28SG805	28SG1509	18SGm1798,28SGm926	LSU5Gm1089	
Me-Eh-LSU-U1018	LSU-U1018	snR40	-	-	-	Y
		28SU 898				
Me-Eh-LSU-G1028	LSU-G1028	snR60	U80	-	TB9Cs2C5	YHT
		28SG 908	28SG 1612		LSU5Gm1192	
Me-Eh-LSU-A2333	LSU-A2333	-	-	PFS14	-	P
				28S Am2551		
ACA-Eh-SSU1315	SSU1315	snR83	ACA4	Pfa ACA 40	-	YHP
		18SU 1290	18SU 1347	SSU1391,1443		
ACA-Eh-SSU626	SSU626	snR161	unknown	-	-	YH
		18SU 632	18SU 681			
ACA-Eh-SSU461	SSU461	snR189	-	-	-	Y
		18SU 466				
ACA-Eh-LSU3008	LSU3008	snR46	ACA16	Pfa ACA 41	-	YHP
		28SU 2865	28SU 4412	LSU3226,3399		
ACA-Eh-LSU1172a	LSU1172	snR81	ACA7	-	-	YH
		28SU 1052	28SU 1779			
ACA-Eh-LSU1172b	LSU1172	snR81	ACA7	-	-	YH
		28SU 1052	28SU 1779			
ACA-Eh-LSU3087	LSU3087	snR37	ACA10	Pfa ACA 32	TB9Cs2H2	YHPT
		28SU 2499	28SU 4491	LSU3305,3478	LSU3psi1336	
ACA-Eh-LSU1159a	LSU1159	-	HBI-115	-	-	H
			28SU 1766			
ACA-Eh-LSU2700	LSU1159	-	HBI-115	-	-	H
			28SU 1766			
ACA-Eh-LSU1080	LSU1080	snR8	ACA56	-	-	YH
		28SU 960	28SU 1664			
ACA-Eh-LSU2997b	LSU2997	-	ACA21	-	-	H
			28SU 4401			
ACA-Eh-LSU1123	LSU1123	snR5	ACA52	-	-	YH
		28sU 1004	28sU 1731			
ACA-Eh-LSU2288	LSU2288	-	ACA27	-	-	H
			28sU 3694			
ACA-Eh-LSU1159b	LSU1159	-	HBI-115	-	-	H
			28sU 1766			
ACA-Eh-LSU2997a	LSU2997	-	ACA21	-	-	H
			28sU 4401			
ACA-Eh-5.8S80b	5.8S80b	Pus7p	U69	-	-	YH
		5sU 50	5.8sU 69			
ACA-Eh-SSU1216	SSU1216	snR35	ACA13	-	-	YH
		18sU 1191	18sU 1248			
ACA-Eh-SSU299	SSU299	snR49	-	-	-	Y
		18sU 302				
ACA-Eh-SSU1212	SSU1212	snR36	ACA36/36B	-	-	YH
		18sU 1187	18sU 1244			
ACA-Eh-LSU2335	LSU2335	snR191	U19/19-2	Pfa ACA 35		YHP
		28sU 2258	28sU 3741	LSU2553,2676		
ACA-Eh-LSU2268	LSU2268	snR32	unknown	-	TB10Cs3H2	YHT
		28sU 2191	28sU 3674		LSU3psi397	

**Note:** snoRNA of *E. histolytica* and its homolog in yeast (Y), Human (H), *P. falciparum* (P) and *T. brucei* (T) is shown with their conserved modification sites.

All of the predicted *E. histolytica* snoRNAs possessed conserved structural motifs characteristic of each class. Secondary structure of the predicted H/ACA snoRNAs was determined by ACASeeker. All of the predicted 58 H/ACA snoRNAs adopted the consensus folding pattern as shown using VARNA: Visualization Applet for RNA [31]. A representative of H/ACA snoRNA is shown in Additional file 3: Figure S3 A. As expected the H/ACA box snoRNAs formed hairpin-hinge-hairpin-tail structure with H box lying in hinge region and ACA box at 3' tail region. Unlike ACASeeker, the C/D box prediction tool did not provide the secondary structure information. Therefore the secondary structure of C/D box was predicted with RNA fold (rna.tbi.univie.ac.at/cgi-bin/RNAfold.cgi) and structures were drawn using VARNA: Visualization Applet for RNA. Secondary structures obtained for C/D box snoRNAs were similar to the published structures for these RNAs (Additional file 3: Figure S3 B).

The genome sequence of other *Entamoeba* species is now becoming available. We checked these data bases to look for close matches to the predicted snoRNAs of *E. histolytica*. Of the 58 predicted H/ACA snoRNAs we found 36 in *E. dispar* and 47 in *E. nuttalli*, while of the 41 predicted C/D box RNAs we found 33 in *E. dispar* and 36 in *E. nuttalli*. There was a high level of sequence similarity (77-100%), which was expected with *E. dispar* and *E. nuttalli* since they are very closely related to *E. histolytica*[32]. However when the same analysis was done with a distant species *E. invadens*, which infects reptiles, we found only 1 H/ACA and 2 C/D snoRNAs matching with *E. histolytica*. Although this result could also be a reflection of the quality of sequence assembly, it shows that *E. invadens* has diverged significantly from *E. histolytica*. Sequence comparison of conserved genes, e.g. rRNA genes also shows high divergence between *E. histolytica* and *E. invadens*[33,34].

### Validation of computationally predicted snoRNAs by RT-PCR and northern hybridization

**Figure 2:**
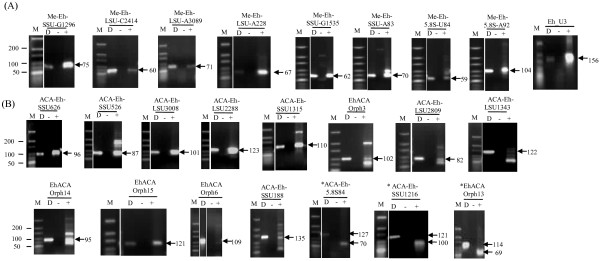
**Expression analysis of *E. histolytica *snoRNAs by Reverse-Transcription PCR (RT-PCR).** 5 μg of total RNA was reverse transcribed followed by PCR with primer pairs specific to each snoRNA. RT-PCR of computationally predicted C/D box snoRNAs (**A**) and H/ACA box snoRNAs (**B**). Arrows indicate the amplicon obtained by RT-PCR. The snoRNAs which have deviated from the predicted size are marked by asterisk. Lane D is the positive control, containing genomic DNA as template. + and – are the RT-PCR reactions with and without reverse transcriptase respectively. Lane M, Size markers 10–300 bp ladder (Fermentas).

**Figure 3:**
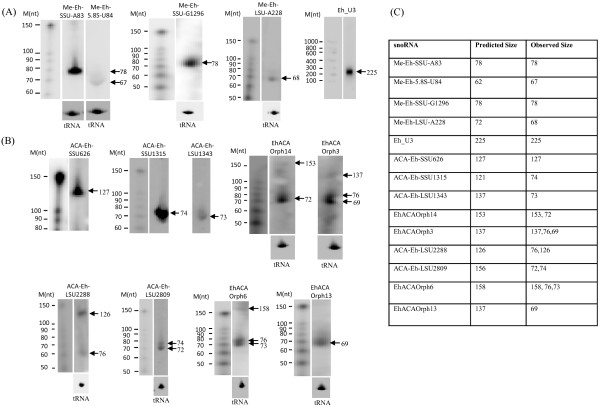
**Expression analysis of *E. histolytica *snoRNAs by northern blotting.** 15 μg of total RNA enriched in small RNA was resolved on a 12% denaturing urea PAGE gel. For Eh-U3 snoRNA 10 μg of total RNA was electrophoresed on 1.2% denaturing agarose. Blots were transferred to nylon membrane and hybridized to P^32^ DNA probe specific to each snoRNA. Northern blot analysis of computationally-predicted C/D box (**A**) H/ACA box (**B**) snoRNAs. The 70 nt tRNA-Glu(AAA) of *E. histolytica* was used as a positive control, as indicated in the lower panel of selected samples. Table displaying the predicted (see Tables 1 and 2) and observed sizes of snoRNAs (**C**). Sizes of bands were marked by end labelled P^32^ decade marker (10 – 150 nt, Ambion).

To demonstrate whether the predicted snoRNAs are indeed expressed in *E. histolytica* cells we selected 24 snoRNAs to represent different categories, namely guide/orphan; and gene location in genic/intergenic regions. Accordingly 8 C/D box guide and orphan snoRNAs were selected (5 intergenic, 1 intronic, 1 in UTR and 1 genic) as also the U3 snoRNA; and 15 H/ACA box guide and orphan snoRNAs were selected (8 intergenic, 7 genic). Expression analysis of these snoRNAs was performed by RT-PCR using total RNA from *E. histolytica* and specific primers for each snoRNA designed from the ends of the predicted snoRNA sequence (Additional file 4: Table S1 for primer sequences). RT-PCR products were obtained for all snoRNAs tested (Figure 2). Amplicons of predicted size (as obtained by genomic PCR with the same primers using total DNA of *E. histolytica*) were observed for all C/D box snoRNAs and most of the H/ACA box snoRNAs. For three of the H/ACA snoRNAs somewhat smaller size amplicons were observed (Figure 2B, marked by asterisk). A possible explanation for this is provided later. To further validate the RT-PCR results northern blot analysis was performed with RNA enriched in small RNA species. DNA probes from four C/D box and nine H/ACA box snoRNAs tested by RT-PCR were used. Results showed detectable bands corresponding to all snoRNAs tested (Figure 3), although intensities of bands were not the same for all, possibly reflecting differential expression levels. For the four C/D box snoRNAs and U3 snoRNA tested, the sizes of observed bands were consistent with the predicted sizes (Figure 3C). However several of the H/ACA snoRNAs showed bands in addition to the predicted sizes. These bands may represent mature snoRNAs obtained after processing, as has been reported in other species [35]. Some of these processing events may involve splicing of internal sequences, resulting in shorter size amplicons in RT-PCR. The multiple bands observed in some of the H/ACA snoRNAs indicate that these may be present as both single and double hairpin RNAs, as is known in other species [36]. On the other hand, northern blot analysis of ACA-Eh-SSU626 indicates the existence of double hairpin H/ACA snoRNA alone in this case; while ACA-Eh-SSU1315, ACA-Eh-SSU1345, ACA-Eh-LSU2809 and ACAEhOrph13 seem to exist as single hairpin alone. Thus, the experimental analysis using RT-PCR and northern blotting demonstrate that the snoRNA predictions by computational analysis are indeed valid and correspond to authentic snoRNA genes.

### Genomic organization of snoRNAs in *E. histolytica*

The genomic location of all snoRNAs (C/D-box, H/ACA-box and orphan) was determined (Tables 1, 2, 3). The majority (69%) of snoRNA genes mapped to intergenic regions, while 20% mapped to protein-coding regions where snoRNAs were encoded either from the opposite strand of the protein coding gene (12%) or from the same strand (8%). A small number of snoRNA genes were located in other parts of protein-coding genes, e.g. in the 5’-UTR (3%), 3’-UTR (3%), and intron (1%). 4% of the genes mapped to non annotated regions (Additional file 5: figure S4). We checked for proximity of snoRNA genes with protein-coding genes involved in ribosome biogenesis, e.g. ribosomal protein genes and genes encoding nucleolar-localized proteins. A gene was considered proximal if it was found within 1 kb of the snoRNA gene. Of the 68 intergenically-located snoRNA genes, 5 were found close to ribosomal protein genes. Of 20 genically-located snoRNA genes 3 were found close to ribosomal protein genes and 1 was close to the gene for fibrillarin, a component of the C/D box snoRNP, while of 6 snoRNA genes located in UTR 1 was located close to ribosomal protein gene (Table 1, 2, 3). Me-Eh-LSU-U1176a was present close to rRNA methyltransferase gene. Therefore a substantial number of snoRNA genes were physically close to genes of related function. The remaining snoRNA genes were located close to functionally diverse genes, e.g. genes involved in cellular signal transduction, DNA (cytosine-5)-methyltransferase gene, heat shock genes etc. When the genomic location of *E. histolytica* snoRNA genes was compared with that of other organisms, some striking similarities were observed. For example, the H/ACA snoRNA ACA-Eh-SSU1216 is localized to the ORF of a hypothetical protein and encoded from its opposite strand. Interestingly the yeast H/ACA snoRNA snR35, which is homologous to ACA-Eh-SSU1216 is also located in an ORF for a hypothetical protein and expressed form the opposite strand [37]. Like in *E. histolytica*, several of the *Drosophila* snoRNA genes are located in the coding strand of a host gene. It was proposed that in such cases alternative splicing may occur, giving rise to two different RNA species, exhibiting different functions, from the same pre-mRNA; an mRNA translated into a protein, and a small non-messenger RNA (snmRNA) functioning as the snoRNA [35]. A striking feature in *P. falciparum* is that some of the snoRNA genes are located in the 3’-UTRs. This feature was found in *E. histolytica* also, where 3 snoRNA genes were localized to 3’-UTRs. Additionally 3 snoRNA genes were also found in 5’-UTRs- a feature not reported in any other system so far. Although we have not experimentally validated the assignment of snoRNA genes to UTRs, these assignments are likely to be correct since we found that snoRNA genes overlapped with protein-coding region of the gene as well as the UTR. In one case (Me-Eh-5.8 S-U84 snoRNA, which is transcribed from the opposite strand of UTR region of receptor protein kinase gene (EHI_021310) we have validated the presence of this snoRNA by RT-PCR as well as northern blotting.

snoRNA genes in other organisms are known to be present both in single and multiple copies, and some may also be in clusters. In *E. histolytica* we found that 80% of the genes were single copy while the rest were in multiple copies. Our data shows that at least in two instances the snoRNA genes may be present in clusters and may be co-transcribed. 1) The snoRNA genes ACA-Eh-SSU1212 and ACA-Eh-5.8 S84 are 126 bp apart and are transcribed from the opposite strand of EHI_098580 gene. Due to their proximity and presence in the opposite strand of the same gene, it is likely that these two genes may be transcribed together and may exist in a cluster. 2) The four identical copies of ACA-Eh-LSU2997a snoRNA genes (located in Scaffold DS572347) are separated from one another by a sequence of 206–214 bp, which is also identical in the four copies. We tried to locate promoters in the 206–214 bp intergenic region of these snoRNA genes using bioinformatic tools (Promoter2.0 prediction server, neural network promoter prediction) but did not find any promoters. The upstream region of the very first copy of snoRNA may have a promoter but this could not be checked computationally as this region was right at the start of the scaffold. It is possible that these four genes may be co-transcribed as a single unit (polycistronic) and may constitute a cluster.

### Structural features of *E*. *histolytica* box H/ACA and box C/D snoRNAs

H/ACA snoRNAs typically fold into a characteristic hairpin-hinge-hairpin-tail structure in which base-paired stems alternate with single-stranded regions (hinge and tail). The H box is located at the hinge and the ACA box is located at the 3' tail, 3 nt away from the 3' end of the snoRNA [15]. The site for guiding uridine modification of the target RNA is always located 14–16 nts upstream of the H box and/or the ACA box [38,39]. This guide site consists of 8–18 base stretch which is complementary to the target RNA. It is located in an internal bulge or recognition loop in each hairpin and contacts the target RNA containing the unpaired uridine to be modified. Each H/ACA snoRNA can guide the modification of one uridine or two uridines which may be located in the same or different target RNAs. Thus the H/ACA snoRNA may contain only one or both functional loops. In *E. histolytica* all the H/ACA snoRNAs (Table 5) adopted the hairpin-hinge-hairpin-tail structure. Some variations were observed, e.g. in some cases the guide sequence may extend into the adjoining P1 and P2 stems flanking the recognition loop (Additional file 3: Figure S3 A) [40]. Of 43 guide H/ACA snoRNAs in *E. histolytica*, 5 snoRNAs (ACA-Eh-LSU1107a, ACA-Eh-SSU631, ACA-Eh-LSU2288, ACA-Eh-LSU1159b, ACA-Eh-LSU1107b) possessed both the functional antisense regions which can either guide the same or different substrate rRNAs. For example, ACA-Eh-SSU631 is predicted to guide the modification of uridine in 18 S rRNA at 2 different positions, 631 and 1114; whereas, ACA-Eh-LSU2288 can guide the modification of uridine at position 1431 in 18 S and at position 2288 in 28 S rRNA (Table 2). Three H/ACA snoRNAs show potential of directing two pseudouridylations by a single guide sequence (Additional file 6: Figure S5), as has been reported in other organisms e.g. ACA19 in human [41]. It is proposed that RNAs get folded into alternate structures thus targeting multiple sites. Overall we found 41 psi sites guided by 43 H/ACA guide snoRNAs. We also found some sites which may be subjected to both methylation as well as pseudouridylation. In human, U3797 position of 28 S rRNA is subjected to methylation as well as pseudouridylation [30]. Similarly in *E. histolytica*, the residue LSU1176 could be guided by C/D box snoRNAs Me-Eh-LSU-U1176a, Me-Eh-LSU-U1176b and Me-Eh-LSU-U1176c as well as by an H/ACA box snoRNA: ACA-Eh-LSU1176. The target site corresponding to LSU1176 is known to get methylated in *Arabidopsis thaliana* (SnoR41Y C/D snoRNA modifying at 25 S:U1064) and pseudouridylated in *S. cerevisiae* (snR49 H/ACA snoRNA modifying at 25 S:U990) [25,29]. Similarly the 5.8 S84 site could be guided by C/D box snoRNA Me-Eh-5.8 S-U84 as well as H/ACA box snoRNA ACA-Eh-5.8 S84.

**Table 5:** **Sequences of box H/ACA snoRNA genes in *E. histolytica***

	
ACA-Eh-SSU1315	TGCAAGT*CTCCAC*AGATTGACATAAAGAATGTCTTATCT*ACTAAGA*CTTTGCA**AGATTA**AAACAAGTTTTAAACTCACGAGTAATATTGAATATTCGTGTTAATAGGGCTTGGAA**ATA**ATC
ACA-Eh-SSU631	ATAAAGTGGA*AAATTCTA*TGGATGCAAATTTTTTTGCATCT*TTTTTCTTT*TTTGT**AAATTA**TTTAGATG*CATTTT*TTCTTTGCTAATTTTCGTACCCATAAGAAGAAAGAATAACAGAAA*TTTAA*TGTATT**ATA**TTT
ACA-Eh-SSU1727	CTGT*GTTTAAAGT*CCAAAGATCTTCAGTTATTCGAATTGCTTCTTTGG*ATAAT*GAAAGACAGT**AAAATGA**GATTGATGTGAACTGTGGGACAACATTCTTGATGTCACTTTCACAATTCACACCAGTTG**ACA**GTC
ACA-Eh-SSU626	TCCACTTCACAAAAATGACACTCATACAGAAGAGTGTGTTTTGGTATTTGACGTAGTGGA**AGATTA**TTTGCTTAG*TAATTC*TATTGATATGACTATTTCTATCAATC*CTACGA*ACTATGCA**ACA**TCA
ACA-Eh-SSU461	TGACTGAGTATGTATTTTGTTCATTTTGTCATCAGCTTGGATATTATTTGTTTATCATTCGATTT**AAATAA**AATAATAAG*GTGTTGT*GTTATAATTATAGTTAAGATGGATATAATTCATGA*CTATC*ACCTTATTT**ACA**CCT
ACA-Eh-SSU1675	TGCAGTTATCCCCTCGTTTTAATTAGTATTAAAACGAACCATTATTATACTGCA**AAATTA**ATTTGCTTTA*TTTTT*AAGGTTTATTTTACTATATTATTTACCTT*CTATTTTAA*AGCAATAA**ACA**ATT
ACA-Eh-SSU526	GCATAG*TTCGTAG*GATTGATAGAAATAGTCATATCAATA*GAATT*ACTAAGC**AAATAA**TCTTCCACTACGTCAAATACCAAAACACACTCTTCTGTATGAGTGTCATTTTTGTGAAGTGGA**ATA**AAT
ACA-Eh-LSU3008	GGATTT*ATCGAAG*CATTAATATACTGAAGATAGTGATTAATG*TCAAA*TATAATCCA**ATAACA**GTGGGTAAGAACTTATGATAAAAGTTTTATTTCTTTGAATAAAATTTTATTGTATTACTCT**ACA**TTT
ACA-Eh-LSU1172a	TTATTTGTGAAGTGA*TTATT*AATCAGTTTATATAATTGATTT*TAGT*CATATTTAATAA**ATAACA**TTTTTGTATGTTTCACATATTTATAATTCATTCATTTTAATTCATAAGTTAATTTATAAATACATACAAAAT**ACA**TTT
ACA-Eh-LSU1172b	TATATTATATAATGTCATTGGACTTACTTTTAAATTATCAGAGTGGCACAAAGATTTTATATTTATG**ACATTA**GTCAACAAAGATATTGAC*TTATT*TCGTAATTCTATTATTTATGGAATTGTGAT*TAGT*ATCTA**ACA**ACA
ACA-Eh-LSU1107b	AAATAAT*TTTTTATTAAT*ATTGTTTTTATTTAAAAATACATAAAATGTATTTTTAAATTAGAGGAA*ATA*GAAAATATTTAA**AAATAA**ATGAATAAA*TTTATC*GATAATTTAACATAACAGTTTGTTTTGTTTAT*TGGTTTGA*AATTCAA**ACA**TCA
ACA-Eh-LSU1650	TACAC*AATCCAAA*GGATGTACAATTTTTTATTTTATGTCC*ATGTT*AATTGTGTG**AGAGAA**TTCTTGAAATATTGTTTAATTCTTATTGAATTGAAATATTATTTTTCAAGGT**ACA**AAA
ACA-Eh-LSU3087	GGTGCT*CCAGCT*AGGCTAAACTCTTTTAGTTGTAGACCT*CGTT*TAAGATCACCT**AGAGTA**AAAGATATTATGAAAAAAAAAAGAAGACATTATTCAATTAATAATGTTTTAAATTCATAATAA**ATA**AAT
ACA-EH-LSU2791	AAGTTAGAG*TGGAAT*GTTTGTTAAACAAAAAGTAGTTTAAAACTACTTAAAA*TAGTCAA*TTTTTAATTT**AAATTA**ATTGTAGGAGTTGTTGGTTATGTGTTTGAGTAAGTTTAAATTGTTAATTTACTAAAACATGACGAAATCATTTTTTCATA**ACA**AAA
ACA-Eh-LSU3155	GTAGTTCAATTGAAATGATGATAATATTCTCTGTATTCTAATCATTTTATAAATGAAGTGCAG**ACAATA**ATGTTCCAAAGATATCTG*ATCTA*TTGAAATAATGAATTGAATATTTTAATTTGAATTTTAA*TACTTT*TCATATTTTA**ATA**AGA
ACA-Eh-LSU3221	TCTT*TGTTTGATT*CTATTTTACTTCAAATGAGGAAGTGTAATTCATTGAAGTATTGGTATAGA*ATAA*CCATTAAAAGA**AGATAA**ATAATTTTAATCAGACTGTACATTGTTTGAAATAAGGAACATGTGTATTTAATTGGAATATA**ACA**ACA
ACA-Eh-LSU1159a	AAATAAAAACA*ACAA*TAATGTTTATATAACATTCAATAAAATATTT*TGTTG*TTTTTCATTTAA**ATAAAA**TATGTTGTGAAGAATATTTCAAAAAAAGTAGAATTATAGTTGTTTATTTCAAATGAATATGAAATGTTTTTAATAATAA**ATA**ACT
ACA-Eh-LSU2700	TGAGACAGTTTGAAGAATGGACAAATAGAAAGGTAGGAGGTGTATTATTTGATTCGTCTGTCTCTGACTGGAATCAG**AGAACA**TCTGT*TTGTGACA*AAATGTTAATTGGGAAAGAGCATATTTTGTTTGTTATAGAAG**ACA**CAA
ACA-Eh-LSU1080	GCTTTC*CTT*ACAACGGCAAAGACATTTTATTCTTTGTGCAGT*GGGAATA*GAAAGCT**ATATTA**ATATTGGTGCTTTACCCTTGAAAATTTCTTTTAATTTTTAAGTCAAAAACATCAT**ATA**ATT
ACA-Eh-LSU1343	AGATG*GTCAA*AGTTAGTGTTGCACATATGATGATTTTATAAGCAGTCATATGAAGCCGAA*TGAAT*TTATCTAAT**ACATAGA**CTATTATGTATCGCAGCTTAACATCAAAGGTGGAGTTGTGTTATTGATAG**ATA**TAA
ACA-Eh-LSU2997b	GGAGTGAT*AAAGC*GGATTGGTAATAGAATAGTGTTAACAATCT*CGTC*AGAATCTCCT**AGATTGA**TTATTATGTTGTATTTTCCCATGAAAAATGAATTCATTTTATCATTTAAAAAATACAAT**ATA**TTT
ACA-Eh-LSU339	TGCTACATGTGT*TTTTC*CATCTTTTTTTGAAGAGACAAAGGATGA*TTTA*GTATGTAGTA**ATACTA**GTGACAAAGAAAATATAATAAGAGAAATGATTAGGTTATATCCTTTAATATTTAATGTTTGTTGTTCTTTTTCAATT**ACA**AAA
ACA-Eh-LSU1123	TGATTGA*TAGT*TTGATTTGGTTTATTCTGAAAATAAAATGTAGAATTAT*TTATCTT*GTCAA**ACATTGA**TAATCAACCGTGCTTATTCATTGTTTCATATTGATATTCTTAATTTCACATTATCAACGAATGAAACGTGTTGT**ACA**AAC
ACA-Eh-LSU1005	TTTTGAGAATTGAAAATATTTATTAAATATTTATTTTATTCAATAGTAAAGGTTTTTAATTTTCAAAACA**AGAAAA**TAATGTTTGTGATAAAACA*AAGT*CATTTTTCATCCATAAAATGAAAAAGGAG*TTTGC*TACAAAAAA**ATA**GTC
ACA-Eh-LSU1236a	TATGGTGTTAGTGTTTGATAGAAAATTTCTTATTCAATACTTCAAGAATATTATTGGACATTTATTATA**ATAGAA**GAACGTGT*ATT*TGTAAAGATAATAGTATTTTTACTTGTTCTGATGC*AAGTAT*ATGTGTTA**ATA**TAG
ACA-Eh-LSU1236b	TATGGTGTTAGTGTTTGATAGAAAATTTCTTATTCAATACTTCAAGAATATTATTGGACATTTATTATA**ATAGAA**GAATGTGT*ATT*TGTAAAGATAATAGTATTTTTACTTGTTCTGATGC*AAGTAT*ATGTGTTA**ATA**TAG
ACA-Eh-LSU1107a	GCAAAATATAATAAATGGA*AAATTCTA*TGGATGCAAATTATTTTGCATCT*TTTTTCTTT*TTTTGC**AAATTA**TTTAGATGCATTTTT*TCTTTGCTAAT*TTTCGTACCCAGTGTGATATGTCAATGAAAATGGAGA*ATGC*AAAAGAATGA**ATA**ATT
ACA-Eh-LSU2288	AGCATATAC*CTTT*CTCACTATATTATTGTAGCGAGA*CATTCAGAG*ATGCTA**AGAATA**AATGAATATTCATTTAACT*TCTCTT*TTATTACTTTAATGGTTTGAA*AGAA*TAATGAATATTCG**ATA**TCT
ACA-Eh-LSU1159b	TTATGTGAAATTC*GATAA*AATATTTTATTTTTTAAAAAATATTT*TGTTG*TTTTTCATTTAA**ATAAAA**TAATA*TGAT*GAAAATTTTAAAAGTGAAGAAATGAAGTTATTTATTTCAAATGAATATGAAAGGTTTTTT*AATAAT*ATTAA**ATA**AAT
ACA-Eh-LSU2997a	TGTTCCTG*AAAGC*GCAGAGACGCACTAGCGTTGTCTGT*CGTCGC*ATTGGGAACA**ACAGAGA**AGGATGATTCCATAGTGGGTGAGATGGCAATGATGCTGTTTCAATGTGGGATTGT**ACA**GTT
ACA-Eh-5.8S80a	TATAATGTAA*ATACT*GATATGGGGTTTATGAAAACTATAAACAATAT*CATTTTATT*CATTGTGT**AAAGTA**ATAAAACACTTTTAATAATAGTACTAAAGTGAAGGGTATTATTTTAGAATATTATGAAAACTGT**ATA**AAA
ACA-Eh-5.8S80b	ATTTTTATTGATGCAAA*ATATT*TAGCAACATTTTTATTGATGCAAAA*TATTTT*GAAATAAAAAT**AAATCA**TCATTTGATATTATTAATATATTTGATAATAAATTATATTATATATTAAATACATC**ATA**TTT
ACA-Eh-SSU740	ACCTCCAAGACATTTCATACTTAAATTAAAACTTAAAGGAAGTTATGATTCTGATGAAGGTAAATTTGGAGGT**AAAGAA**CAAGGAATATTAGA*ATTT*GATTTCTTTATTAAAACAATTCAA*AGTAATA*TTCCTAG**ACA**TCT
ACA-Eh-SSU188	AGAGA*TGTACT*TAGTATGGATAACATGTATAGTGCATGGAATCCTCAAT*TAC*TTATTTCT**ATAAAA**ACTCCTCAAGCTGTTAATGTTGCCAATGCTTGTACTCTTACCATTCATTGGCAATTTGATGTT**ACA**TTG
ACA-Eh-SSU1216	GTTATGAAGAGGTTCTATTATCTGTTTATCTATTGAATTATAAGGAACTGGTTCAGGAC**AGAGAA**ATGAATCAGTTGGAG*GTGTT*TGTTTTTGGAATTCATTAAATGAAAAGAAGAAATT*GTCA*CAACAGTCTGAA**ATA**AGC
ACA-Eh-SSU299	CCATACGTTCTTTATTGGAGGCAGTCCTTATTTTGTAGAAGAAAATAAGATTTTACTTCAATCTCAAAAAACAAATGG**AAACGA**AGCGGTTTTAATGA*CAGA*AGAAGAGATGAGATCATTTTATGACATTTTTGATTATTTATCTTC*TTCTCA*ATTACAAACC**ACA**AAA
ACA-Eh-SSU1212	TTATCATCATCAAAGAAATTTGTTATTAATGATTGCTTTGGTTGTGATGGCTGC**ATAGGA**ACAGCGACTTG*GTGTTGATT*AAGATAAGGGTTAAAAGTACTTTGTTGA*AATTGAG*GTTGTTGA**ATA**TAT
ACA-Eh-LSU2809	AGTTACTGTGCAATTTTTTGTGGGTTGAACAGTTTTCCAATTCTGATTAATTGTAAGCATAGAACTA**AAACAA**TCATCAACAAGAG*TCATTTCAGAAT*AAACAGTAATAGAGTCACAATCAATTTCTGAATAT*TTG*TTATGTGTTCTTGT**ACA**ATC
ACA-Eh-LSU2335	GATTGAATATTT*TCA*GTTACTTCATGAACATGAGGTTTAGGAGGAATT*GTTACT*ATTTGGTTTA**AAATAA**CAGAATTTATACTTTCTGTTATTGGTTCAAAAAATGAATTTGATGGAAAGATAAC**ACA**TAA
ACA-Eh-LSU2493	GGGCT*TTAGAGTT*GTGTATTTTTTCTTTTAACCAATTTCTACAAATGGTGTGAGCATG*GTT*ATAAAGTTC**AAATGA**AAGTGGAGTAGAGGTGTTGTATTTAATCTTATCAAAATCTACTGCTTTATTTA**ATA**AGT
ACA-Eh-LSU1176	GGTGGATATA*TTGTT*AAGAATAGTTTTGGATACCAACATGGTCATTCATTAAAATATTGGC*TTCA*ACAACATTCTATT**AAAGAA**GAAATGCAAATATGTCCTAATGTACGTTCGTTTGAACAATGGACTCCATTAGATGAGGATTGTATTA**ATA**AAC
ACA-Eh-LSU2268	TACTAAGACAAATTTGTCCATTTGGAAATACATTTGGATGGAAAAATCCTTCTGGTAA**ATAACA**ACGTGGTGGTGATG*CTGGGTA*TCCAGTAGAAAATTTCATTTCTACTGGAT*AAT*AACCATTTTCCC**ATA**TCG
ACA-Eh-5.8S84	GTTTTGAGTTAT*TTTTGAA*GATGATTGTTTATTTTCATTTTCATC*TTCAC*TTTCAAAGCCA**AAATCA**TCGAACACAGAGTTTTTATCTTTTTGTGGTTCTACAAGGGTTGTAGTTTGACTTGAAGGAATAACATTATTTTGACTAG**ACA**ATC
EhACAOrph1	GCATTGCTTTTTTTGATAAATACTTATTTATTTATCTTCGCCGCAATGCA**AGAAAA**TATTTCAATTAGCAGTGCTTTCTTTAAAGGAGGAAATCACGATATAATTGAAG**ACA**TTA
EhACAOrph2	TTTCAAATAGAATTTCCCGGAGAAATACCACAAAAGGGTGTGAAATGGGTTTTTGAAAT**AGAATGA**ACTGATTTATTAAATCAGATATGTCGCTTTCAAACGATGGACGACGTATGAGGGGAGTGAATG**ATA**GAA
EhACAOrph3	TTGTTTTTTGATTAAACCACAATTTTTATAATATGAAAAGATAATTGTGTTTGGACAATTTAAAACGA**AAAGAA**ATAGCGATTTAGGGTAGTTCATTCTATGTAAATATAAATGAACACTATTTAATCGCA**ATA**TTA
EhACAOrph4	GCAAAGGGTTAGTATTTTATTTAGTTATTGAAATTAGATAAAAACACCCTGTGCA**AGACAA**ACGTGTAGATCCTAATAAAGAGAAGTCTTGTCTATTTCTTTTTATCTGTCTACAA**ACA**AAT
EhACAOrph5	TGAGACTCTACGGTTATTAATTTATATGAATTAATAATAACCGAGTTCTCAA**ACAGAA**AATAATCATATAAGGTATATAAAATAATAACAAATAAGATGTTATGATAATTAGATTATATGATA**ATA**ATT
EhACAOrph6	GACATGCCATAAACAATGTTTTGTATAACATTTACGACTATCATCATAAATGTTTTATAAAACACTCCGTGTC**ACAGTA**TTAAAGTGACCGTAATGTTAGGGAAGTTTCCCGAAAAGTAGGGACAACAAATCCCTAACGACAAAGGTGTCAC**ACA**AGT
EhACAOrph7	GTCATCCCTTCAGATCATGGAATTACATTCAACACTAATCTGGGAGATGATGACAA**AAATAA**TGTCATTGAGGAGCATGATTCATTTGAGTCTGTTGAATATCTTTATGATCGTAATCTCGATG**ATA**ATC
EhACAOrph8	CCAAATAACAAAAAGAAGAGCATTAATTAGAAAGAAAAAGAATGACTAAGGTTATTTGGT**AAATTA**ATAGTGATAAAAGGAAACATAGTTCAAAAGAGGAGTGAGCTATGTGATTGTTTAACACA**ACA**AAG
EhACAOrph9	GCAAATGATATTCGTATATCAATTTTCAAGTTAATTGATTTGTTATTGTTTGCG**AGAAAA**ATTAAAGATAGAAGTTATTTATATCTTTTGGTATAAATAAAAGAGAATCTTTGA**ACA**TTA
EhACAOrph10	ATTAGAAGTAAAGTGAGGATAACTTAATAACTCTGTTGTTCTTATTTGTATTGAGTTGGTCAAC**AGATAA**CAATGGACAATTATAATATAAACATTTTATTATATTTGGTGTTTCTAATTTAAATAAAATGTTACATTGTTGA**ACA**ATT
EhACAOrph11	TTGGATTTAATTGTACATTATGTCCAGCTTGTTGAGTTAAATCTGGCAGTGGAATAAGTCCAAC**AGATAA**TAAGAGACAATCACACTCAATTTCATATTCTGTTCCTGCAATTGGTGCAAGTGTCTTTGGATC**ACA**TTT
EhACAOrph12	GGTTTATCATCTTCAAATCCAATGGCTGATGCTATTTCTTTGATTTGGTTAAAAGACTC**AAAATA**TTCTTCTTCGACAAATTTTGATTGTTCATCTAATTGATGTTTTAATTCTAAAATTTGTTGAAT**ATA**ACC
EhACAOrph13	ATTATTTTGGATAATGCTAATGTTGATTTACAGGATGTTATTCGTGATAATGTG**AAAATA**AAAGTTCATGTTGGTCGTGGTATTGTAGTTGGAGGATTTCAGGGATCGGATGCCGCGGATGTTGAAGCTGC**ATA**TAA
EhACAOrph14	ATCATTAGAACATGTAAATGATGATAGTTCTGTGTCAGAAACACCAAACATCCCTTTTACTTTAGCTGATGATA**AAACCA**ATTCAATAACTAGTGAAATAGCTTTTTGTTGTTTATTATAATAATATTTATCACTAATACCATTGAA**ACA**AAA
EhACAOrph15	GTAGTGGAACAATAAAATGACTATTAGGTAGTGATAGATAGTCATTATCATCAATAATTATTTTCTCTATTACT**ACAGCA**CTATTTAATATTTGTAATTCTACAGAAGTTTCATTTTTCTTAAGAGTATAAAGAAAAGGTGG**ATA**ATG

**Note:** Box H and box ACA are depicted in bold. Antisense elements are in italics.

The C/D box snoRNAs typically possess the conserved boxes C (RUGAUGA) and D (CUGA) near the 5' and 3' ends, respectively [1]. A short region upstream of C box and downstream of D box usually shows base complementarity. Base-pairing in this region brings the C and D boxes close together. In addition to C and D boxes, some snoRNAs of this class also possess C' and D' boxes which are less conserved and form a folded structure in the order 5’-C/D'/C'/D-3’. The 2'-O-ribose methylation of the target RNA is guided by one or two 10-21nt antisense elements located upstream of the D and/or D' boxes in a manner such that the modified base is paired with the snoRNA nucleotide located precisely 5nts upstream of the D or D' box [3,4]. All 41 C/D box snoRNAs in *E. histolytica* had the conserved motifs: C box and D box. The C box had the consensus sequence RUGA [U/g/c/a]G[A/u]. The sequence of D box in two of the C/D box snoRNA genes Me-Eh-LSU-U3580b and Me-Eh-SSU-U871 was AUGA. All of the other snoRNA genes possessed the consensus CUGA sequence in the D box. 71% of these RNAs possessed the D’ box as well (Table 6). The D' box is much less conserved and it varied from CUGA to CAGA, UUGA, AUGA, ACCA and CCGA. All the C/D box snoRNAs possessed at least one antisense element upstream to either the D’ box or D box. Me-Eh-SSU-A1183 snoRNA gene had two antisense elements and was able to guide different target sites of the same or different rRNAs (Additional file 7: Figure S6A) whereas Me-Eh-SSU-G1535 and Me-Eh-SSU-A790 had single antisense element upstream to D’ box which could guide multiple sites for methylation in different rRNAs (Additional file 7: Figure S6B (i-ii)). Five C/D box snoRNAs with a single antisense stretch in each were predicted to target different sites in the same target RNA (Additional file 7: Figure S6C (i-v)). From the predicted folding pattern 60% C/D box snoRNAs possessed the terminal stem while the rest either lacked it or had an external stem, or an internal stem [42].

**Table 6:** **Sequences of C/D box snoRNA genes in *E. histolytica***

	
Me-Eh-SSU-G1296	TGTA**ATGATGA**GATTTTA*CCATGCACCACT****CAGA***ATTATCTACCCAAAGATAAGTTGTGTTGATTATGGTGT**CTGA**AC
Me-Eh-SSU-U1024	CACT**GTGATGA**AGCT*TTTTATCCAATCCT****CTGA***ATATCGTTGATATTTATCTATGTGGATATTAATGTTGACTT**CTGA**GT
Me-Eh-SSU-A83	GAAG**ATGATGA**CTAGACTTGGCAGTCTCC*CTGTTCGCAGTTTCAT*A**CTGA**ATAAATATGAGGATAAAGGGTT**CTGA**TT
Me-Eh-SSU-G41	AGAA**ATGATGA**CTTGTGT*GCTTAATCTTT*G***TTGA***TTCAAAAATGATAACACTTCTTTAAAGT**CTGA**TT
Me-Eh-SSU-A431	GCAA**ATGAGGA**AATAAAATT*TGGGTAATTTACG*T***CTGA***AATTGATGATAACCATCTGTCGTT**CTGA**TG
Me-Eh-SSU-U871	AACG**ATCATGA**AT*TTTCACCTCTCCCGTTTTTT*T***CTGA***ATCACCCCAATTATTCCTTTTAATCCTTCTCTCGAA**ATGA**TT
Me-Eh-SSU-G1535	TCGA**GTGACGA**TAAAC*CACAGACCTGTT****CTGA***CCTTAATGGAGATAACAGAGCTGGCTCCAATTAGCGCTGGGGCT**CTGA**CG
Me-Eh-SSU-A27	GTCA**GTGATGA**TCAATAA*ATCAGCATATA*T***CTGA***ATAAAGTATGATGGTTTAAGACGGGT**CTGA**GA
Me-Eh-SSU-A1830	CAAT**ATGATGA**AAAAGCACCAAC*TCACCTCTTTA*G***ATGA***TATTCCTGATTTTGATTTTGATGAAATGATTAACCAAA**CTGA**GG
Me-Eh-SSU-A836	CTTT**TTGATGA**ATAAACT*CTTTTAATCTTTCT*T***TTGA***ATTTTCTTTTCTCTTTTTCTTTCTTTTGAATTTTCTTCTAACTTTTCTTTTAGAGGCTTG**CTGA**GG
Me-Eh-SSU-G1152	GGTA**ATGATGA**TAGAAAGTTTTCAGATTATTAATGAAGACAT*TTTCAGCCTTGT***CTGA**GC
Me-Eh-SSU-G628	TAAA**ATGATGA**TTATAG*TTTTAATACAAC*A***TTGA***TTTAAATGAAACACACAACTTTCACTAATTTTAATAATCTAATTTTTACAATTAACT**CTGA**CT
Me-Eh-SSU-A1183	AAAA**ATGATGA**AAAAAGAAAAAAG*TCCTGGAGTTCC*A***ACCA***GGATGAATATCCATGATGATAAACTAATCTTCTCA**CTGA**TT
Me-Eh-SSU-A790	AGAA**GTGATGA**TATATAAATT*CCATGTTAGAA****CTGA***TATAACGTGTTGATATTTGTATAAGT**CTGA**TC
Me-Eh-SSU-C1805	GTAG**ATGATGA**CTTATA*CGTCGGGCGG*A***CTGA***AAGATTATATGTAGATTCGACGTGT**CTGA**TA
Me-Eh-LSU-A928a	ACCA**ATGATGA**TTTACATTAA*ACCATCTTTCG*T***CTGA***AAAACTGATGTCAAATATGTCATAAT**CTGA**GG
Me-Eh-LSU-A928b	TAAG**ATGATGA**TTTGAT*TCCGTGTTTCG*T***CTGA***ATCCTGGTGAAAACTCGACAATCTTAT**CTGA**TT
Me-Eh-LSU-U1868	TTCT**ATGATGA**TATTTAATGA*AAGAAGAAAAGAG*T***ATGA***ACTTAACTCAAAAAAATATAACGGTGGTGCTTTACCTAAAATCTCTTTTTTTCGTC**CTGA**AT
Me-Eh-LSU-U3580a	GAAT**ATGATGA**AGTATTTTAATAAGAAATAT*AATAAATAATAATAGAAAGA****ATGA***AATAAGATAATATGAAAGAATAAGAAAAATAAAAAGATATAA**CTGA**TG
Me-Eh-LSU-U3580b	GAAT**ATGATGA**ATTAATTTAATAAGAAATAT*AATAAATAATAAAAGAAAGA****ATGA***AATAAGATAATATGAAATAATAAGAAAATAAAATGATATAAATG**ATGA**TA
Me-Eh-LSU-A785	AGAA**ATGATGA**TAATG*TGGTCCGTGTTT****CTGA***ATACTGAAGAGACTATAACCACTT**CTGA**TT
Me-Eh-LSU-G2958	AGCA**ATGAAGA**TATACG*CAGTTATCCCTGT****CCGA***GAACTGCAAATGTGGATATGTTAACTAAGT**CTGA**GC
Me-Eh-LSU-A3089	AGAA**ATGATGA**AATAAT*ACTCAGCTCAC*T***CTGA***ATATAAATGAAGAATGAGTTTCTATATGATTT**CTGA**TT
Me-Eh-LSU-C2414	GTCT**GTGAGGA**ATTGAA*AGATAGGGACA*T***CTGA***TATAACTGATGTTAAAAATCTTTGATTTGA**CTGA**GA
Me-Eh-LSU-G926	TGAA**GTGATGA**TCCTTTATTTAAGTGATTAACCATGATAAT*CATCTTTCGGGT****C*TGA**TT
Me-Eh-LSU-U1018	GAAT**ATGATGA**ACTTAATCA*ATATTCAAATA*G***CTGA***ATAATATGATAAAATGAAAGTCTGTTA**CTGA**AA
Me-Eh-LSU-G1028	TATG**ATGATGA**AATGAGTCTCCGAATAATATTGAGGACAAA*TCTTTCGCTCCTAT***CTGA**TT
Me-Eh-LSU-U1176a	TATA**ATGATGT**ATATTTTCTTCATTAACAAT*TTCTTTGTTTATTTA****TTGA***ATTTAGTTGATAATTCATTATTAACACTACAACAACGTTTTGAATATCTTTTA**CTGA**AG
Me-Eh-LSU-U1176b	TATT**ATGATGT**ATATTTTATTCATTAACAAT*TTCTTTGTTTATTTA****TTGA***ATTTAGTTGATAATTCATTATTAACACTACAACAATGGTTTGAATATCTTTTA**CTGA**AG
Me-Eh-LSU-U1176c	TATA**ATGATGT**ATATTTTCATCATTAACAAT*TTCTTTGTTTATTTA****TTGA***ATTTAGTTGATAATTCATTATTAACACTACAACAACGTTTTGAATATCTTTTA**CTGA**AG
Me-Eh-LSU-A2333	TGTA**ATGATGA**GAACTTTATGAATAATAGAGAGGATTCTTATAAAAAGAAGTGGTAATATTCTCGTTTTGAAAATGTTACCAGGGATGAATAATCTCCCTTGATGATTCTT*TCATAGTTACT****C*TGA**AC
Me-Eh-LSU-A228	ACAT**ATGATGA**ATTTCTTGGA*GAACTGAATTTAAA****TTGA***AGACAATTTATATTATGTTGCAAAGAA**CTGA**TG
Me-Eh-5.8 S-U84	TATA**ATGATGA**TATAAAACAATAAATTATGACTTTTC*TTCAATTTTTTGATATTCA****C*TGA**AA
Me-Eh-5.8 S-A92	TGTA**GTGATGA**TGGAAGAATTA*ATTCAAATTTT*A***ATGA***ATTAGTGTTATATACTGAAAGAGAGAGAATAGATGAGTATTGTGAAAGGTCTAACCTTCCTTTAAATACTA**CTGA**AA
EhCDOrph1	CTAAATGATTTTCTAA**ATGATGA**CTCTTGTGGTGGTTTTGGAGAAGACTGATTTGATGAATAAGAAGATGACCATC**CTGA**AGAACATTCATTTGG
EhCDOrph2	GACTTGATAGAATTAA**GTGATGA**CATGTGTTGAACAATCTCTGAGTTTTGATGACAACTTACCTTCGT**CTGA**TATTTCTTTTTCTTC
EhCDOrph3	AATTAAAAAAATAACA**GTGATGA**CTTTACTGCGTTATCTTAAGTAGGATTCTTTTATAGTTTCCAGTGATTTCAACTTTCACTTGAGT**CTGA**GTTATTCTTTTTATA
EhCDOrph4	TTTAATCAAATCCACA**GTGATGA**AATAACTTGTCTGAGAGTCATTTTTAATCATGATGGCATGTTTTTATTT**CTGA**GTGGGTTATTTAACT
EhCDOrph5	ATAATAAGATGTAAGA**ATGATGA**AGTTTTTATTAAACTATGAATATTACATGATTACTTGATCCT**CTGA**CTTACATTTAATTTT
EhCDOrph6	TTTGAATTAGAAGACG**ATGATGA**ATTTGAATTAGAAGACGACGAAGAAGAAGATGATGAATAAATCCTTAAATAA**CTGA**GTGCTTATATTCAAA
EhCDOrph7	TTTGAATTAGAAGACG**ATGATGA**ATTTGAATTAGAAGACGACGAAGAAGAAGATGATGAATAAATCCTTAAATAA**CTGA**GTGCTTATATTCAAA

**Note:** Box C and box D are depicted in bold. Box C’ and D’ represented in bold and italics. Antisense elements are in italics.

### Computational identification and validation of multiple copies of U3 snoRNA in *E. histolytica*

**Figure 4:**
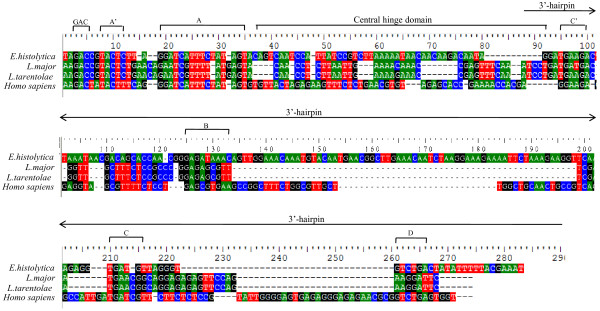
**Sequence alignment of Eh_U3 snoRNA.** Alignment of Eh_U3 snoRNA sequence with U3 snoRNA of L. major [GenBank: NC_007264, complement (226475–226617)], L. tarentolae [GenBank: L20948] and H. sapiens [GenBank: X14945] is shown. The conserved boxes GAC, A', A, C', B, C and D along with central hinge domain and 3’-hairpin is shown.

U3 snoRNA belongs to the C/D box snoRNA category and performs the specialized function of site specific cleavage of rRNA during pre-rRNA processing. It is present in all eukaryotic organisms either as a single copy or in multiple copies [43]. BLASTn analysis of yeast and human U3 snoRNA with *E. histolytica* whole genome revealed the presence of 5 copies of U3 snoRNA (Eh_U3a-e) in *E. histolytica.* These were 97-99% identical to each other and ranged in size from 209–225 nt. All copies were located in intergenic regions (Table 7A) and their sequences are given in Table 7B. The characteristic boxes- box GAC, A’, A, C, B, box C and box D of *E. histolytica* U3 snoRNA were conserved (Figure 4) when compared with U3 snoRNAs of selected organisms (*H. sapiens*, *Leishmania major* and *Leishmania tarentolae*). The Eh_U3 snoRNA was well conserved with respect to *T. brucei* and *T. cruzi*[43]*.* However, it showed poor homology with *P. falciparum* U3 snoRNA [21]. Sequence conservation was greater at 5’ end up to central hinge domain, with less conservation in the 3’ hairpin region. We checked for the conservation of U3 snoRNA among *Entamoeba* species and found 6 copies of U3 snoRNA with 91% identity in *E. dispar* (Table 7A) and 1 copy with 96% identity in *E. nuttalli*. No homology was observed for *E. invadens*. To validate the predicted U3 snoRNA in *E*. *histolytica* we did RT-PCR and northern blotting with total RNA (Figure 2A, 3A). RT-PCR was performed using specific primers for U3 snoRNAs (Additional file 4: Table S1). The predicted and the observed sizes as obtained by both RT-PCR and northern were the same. The sequencing of one of the clones of the RT-PCR product confirmed the presence of Eh_U3e copy of U3 snoRNA.

**Table 7:** **U3 snoRNA genes in *E. histolytica***

**U3 snoRNA genes**	**Len (nt)**	**Seq (%)**	**Scaffold**	**Start**	**End**	**Homology Yeast/Human**	**Location**
A. U3 snoRNA genes							
Eh_U3a	209	91%	DS571856	3136	3344	snR17a/U3 U3	IR
Eh_U3b	225	92%	DS571750	1819	1595	snR17a/U3 U3	IR
Eh_U3c	221	91%	DS571479	13861	14081	snR17a/U3 U3	IR
Eh_U3d	221	91%	DS571353	16563	16343	snR17a/U3 U3	IR
Eh_U3e	225	91%	DS571336	2559	2783	snR17a/U3 U3	IR
B. Sequence of U3 snoRNA genes							
Eh_U3a	TAGACCGTACTCTTAGGATCATTTCTATAGTACAGTCAATCCATTATCCGTCTTAAAAATAACAACAAGACAATAGGATGAAGACTAAATAACCAACAACACCAACGGGAGATAAACAGTTGGAAACAAATGTACAATGAACGGCTTGAAACAATCTAAAGAAAGAAATTTCTAAAGATGGTTCAAGAGGTGAATGTTAGGGTGTCTGA						
Eh_U3b	TAGACCGTACTCTTAGGATCATTTCTATAGTACAGTCAATCCATTATCCGTCTTAAAAATAACAACAAGACAATAGGATGAAGACTAAATAACCAACAACACCAACGGGAGATAAACAGTTGGAAACAAATGTACAATGAACGGCTTGAAACAATCTAAAGAAAGAAATTTCCAAAGAAAGTTCAAGAGGTGATGTTAGGGTGTCTGACTATCTTTTTATGAAAT						
Eh_U3c	TAGACCGTACTCTTAGGATCATTTCTATAGTACAGTCAATCCATTATCCGTCTTAAAAATAACAACAAGACAATAGGATGAAGACTAAATAACCGACAGCACCAACGGGAGATAAACAGTTGGAAACAAATGTACAATGAACGGCTTGAAACAATCTAAGGAAAGAAATTTCCAAAGAAGGTTCAAGAGGTGATGTTAGGGTGTCTGACTATCTTTTTATG						
Eh_U3d	TAGACCGTACTCTTAGGATCATTTCTATAGTACAGTCAATCCATTATCCGTCTTAAAAATAACAACAAGACAATAGGATGAAGACTAAATAACCAACAACACCAACGGGAGATAAACAGTTGGAAACAAATGTACAATGAACGGCTTGAAACAATCTAAAGAAAGAAATTTCTAAAGATGGTTCAAGAGGTGATGTTAGGGTGTCTGACTATCTTTTTATG						
Eh_U3e	TAGACCGTACTCTTAGGATCATTTCTATAGTACAGTCAATCCATTATCCGTCTTAAAAATAACAACAAGACAATAGGATGAAGACTAAATAACCGACAGCACCAACGGGAGATAAACAGTTGGAAACAAATGTACAATGAACGGCTTGAAACAATCTAAGGAAAGAAAATTCTAAAGAAGGTTCAAGAGGTGATGTTAGGGTGTCTGACTATATTTTTACGAAAT						

**Note:** “Len.” denotes length of the snoRNA genes; “Seq.” is sequence identity of corresponding snoRNA genes in *E. dispar* and “IR”, intergenic region.

## Conclusion

Ribosome biogenesis in eukaryotic cells requires the activity of a highly conserved set of small RNAs, the snoRNAs. In this study we show that the parasitic protist, *E. histolytica,* thought to be an early branching eukaryote, possesses the major classes of snoRNAs as judged by sequence conservation with yeast and human. These RNAs are expressed at fairly high levels as they are readily detectable by northern blots. It is relevant to ask whether *E. histolytica*, being a human parasite, has evolved any snoRNA features uniquely shared by other parasitic protozoa infecting humans. Amongst these organisms, studies on snoRNAs have mainly been reported with *P. falciparum* and *T. brucei*. When the features of *E. histolytica* snoRNAs are compared with these organisms, the following points emerge. Both in *P. falciparum* and *E. histolytica* some snoRNA genes are located in the 3’- UTR, a property not reported in any other organism except *Drosophila*[35] where an H/ACA-like snoRNA is reported to be present in 3’ UTR. In addition, some *E. histolytica* snoRNA genes are also found in the 5'-UTR which is unique to this organism so far. Both in *P. falciparum* and *E. histolytica* most (80%) snoRNA genes are present in single copy whereas in *T. brucei* most of the snoRNA clusters are repeated in the genome with few clusters carrying single copy genes [19]. The clustering of snoRNA genes is frequent in *P. falciparum and T. brucei.* We have reported two instances in *E. histolytica* where these genes may be clustered. Unlike *P. falciparum* where 9 snoRNA genes are found in introns, we could locate only one snoRNA gene in an intron, while the majority of them were in intergenic regions, whereas no intronic snoRNA has been reported in *T. brucei* so far. Like *T. brucei*, *E. histolytica* also possesses single hairpin H/ACA snoRNAs which are likely to be processed from a double hairpin pre-H/ACA snoRNA into single hairpin snoRNAs, whereas in *P. falciparum* single hairpin H/ACA snoRNA has not been reported. Unlike *T. brucei* which possesses H/AGA box [36], both *P. falciparum* and *E. histolytica* contain the highly conserved H/ACA box. In contrast to *P. falciparum* and *T. brucei* where the number of methylation sites is much larger than psi sites, in *E. histolytica* we find an almost equal number of both kinds of modifications. There are 47 methylation sites and 41 psi sites. In overall sequence, *E. histolytica* snoRNAs are much more homologous to yeast and human than to *P. falciparum* and *T. brucei.*

The greater sequence homology of *E. histolytica* snoRNAs with yeast and human compared with the two parasite species, and the lack of any particular snoRNA features unique to all three parasite species shows that this highly conserved RNA modification machinery is unlikely to be linked to pathogenesis and each parasite species has evolved its own distinct snoRNA features. This study will help to further understand the evolution of these conserved RNAs in diverse phylogenetic groups and will be very useful in future studies on pre rRNA processing in *E. histolytica*.

## Methods

### Extraction of putative methylation and pseudouridylation sites in rRNA of *E. histolytica*

We used the known methylation and psi sites of five different eukaryotic organisms: *A. thaliana, C. elegans, D. melanogaster, S. cerevisiae* and *H. sapiens* to find putative methylation and psi sites in *E. histolytica* rRNA (5.8 S, 18 S and 28 S) [25]. Alignment of rRNA of *E. histolytica* and selected five organisms was carried by EMBOSS pair wise alignment tool separately (Additional file 1: Figure S1). This gave us putative 173 methylation and 126 psi sites.

### Search for *E. histolytica* C/D box snoRNAs

Snoscan and CDSeeker were used to score potential guide and orphan C/D box snoRNAs respectively from the whole genome sequence (WGS) of *E. histolytica*. WGS was downloaded from ncbi [NCBI:AAFB00000000] (updated on April 17, 2008). The tools were initially used with this file and the results obtained were checked periodically online with the updated genome file. Snoscan is based on the greedy search algorithm. It identifies six features in the genome: box C, box D, a region of sequence complementary to target RNA, box D' if the rRNA complementary region is not adjacent to box D, the predicted methylation site based on the complementary region and the terminal stem, if present [23]. CDSeeker can be used to find both guide as well as orphan C/D box RNA but in the present study it was used to find orphan C/D box snoRNAs in *E. histolytica*. The CDSeeker program combines probabilistic model, conserved primary and secondary structure motifs to search orphan C/D snoRNAs in whole genome sequence. It searches for same features described for snoscan but for the search of orphan C/D box snoRNAs it looks for predicted conserved functional region next to box D or D' (if D' is present) [24]. Both the tools need genomic DNA sequence and rRNA sequences as an input requirement (optional for CDSeeker). All hits that had scored higher than 14 bits were selected as positive guide C/D box snoRNAs [26]. For orphan C/D box snoRNAs, score was set to be 18 bits. These threshold values given are those used for *S. cerevisiae* (for guide snoRNAs) and the default value used in CDseeker (for orphan snoRNAs). BLASTn analysis of predicted snoRNAs with EST database of *E. histolytica* revealed the authenticity of predicted snoRNAs. To find the homology between closely related species *E. dispar*, *E. nuttalli* and *E. invadens*, we did BLASTn analysis of selected snoRNAs with WGS of *E. dispar* SAW760 (NCBI: AANV02000000) *E. nuttalli* P19 (AGBL01000000) and *E. invadens* IP1 (NCBI: AANW02000000).

### Search for *E. histolytica* H/ACA box snoRNAs

ACASeeker was used to screen out potential guide and orphan H/ACA box snoRNAs similarly as mentioned above for CDSeeker. ACASeeker program combines probabilistic model, conserved primary and secondary structure motifs to search orphan and guide H/ACA snoRNAs in whole genome sequence. It identifies following features common for both orphan and guide H/ACA box snoRNA genes: box H, box ACA, hairpin 1, hairpin 2, and hairpin-hinge-hairpin [24]. For guide snoRNA genes, another feature: two regions of sequence complementary to target RNA in a hairpin, was taken into account. This tool needs WGS and the list of putative psi sites (optional) as an input requirement. We have provided the list of putative psi sites (as obtained in method section 1) thus 186 guide H/ACA snoRNAs were predicted on the basis of putative sites and 475 snoRNAs with no putative sites were predicted as orphan H/ACA snoRNAs. The threshold value was 40 bits and 27 bits for H/ACA guide and orphan snoRNAs respectively, which was the cutoff used to train the software SnoSeeker on vertebrate snoRNAs. The snoRNAs were further analyzed for genomic localization in intron, intergenic region or from the ORF of protein coding genes*.* BLASTn analysis of predicted snoRNAs with EST database of *E. histolytica* revealed the authenticity of predicted snoRNAs. To find the homology between closely related species *E. dispar*, *E. nuttalli* and *E. invadens*, we did BLASTn analysis of selected snoRNAs with WGS of *E. dispar* SAW760 (NCBI: AANV02000000) *E. nuttalli* P19 (AGBL01000000) and *E. invadens* IP1 (NCBI: AANW02000000).

### Validation of snoRNAs by RT-PCR and northern hybridization

Total RNA was isolated from mid log phase trophozoites (~ 5x10^6^cells) using Trizol reagent (Invitrogen) as per manufacturer's instruction. DNase I (Roche)-treated RNA sample (5 μg) was reverse transcribed at 37°C using MMLV (USB) with specific reverse primers (Additional file 4: Table S1) as per protocol prescribed by manufacturer, followed by PCR with forward primers. PCR with genomic DNA was used as control. Oligonucleotides used for RT and RT- PCR reactions are listed in Additional file 4: Table S1. For northern analysis total RNA and total RNA enriched in small RNA from ~ 5x10^6^ cells was isolated using trizol (invitrogen) and miRNA isolation kit (Ambion) respectively as per manufacturer's instructions. 15 μg of total RNA enriched in small RNA was resolved on a 12% denaturing urea PAGE gel. For Eh_U3 snoRNA 10 μg of total RNA was electrophoresed on 1.2% denaturing agarose and transferred to Genescreen plusR membrane (Perkin Elmer). Probes were prepared by random priming method (NEB blot kit). Hybridization was carried out in buffer (1 M NaCl and 0.5% SDS) at 42°C for 36 hrs. Post hybridization washing of membrane was done as per instructions suggested by manufacturer. Blot was exposed for 48 hrs in imaging plate of phosphorimager for autoradiography.

## Competing interests

The authors declare that they have no competing interests.

## Authors’ contributions

SB proposed and designed the research, drafted the final version of the manuscript, AB designed and analyzed the computational work. DK and RS performed the computational work. AKG and VK performed the experiments regarding RT-PCR and Northern blotting. All authors have participated in preparing the manuscript. All authors have read and approved the final manuscript.

## Supplementary Material

Additional file 1 **Figure S1.** Global alignment of lsu rRNA of *S. cerevisiae* and *E. histolytica* to predict the putative modification sites in *E. histolytica.* Red and yellow dots are already known methylation and pseudouridylation sites of *S. cerevisiae* respectively. Blue and green dots are the putative methylation and pseudouridylation sites of *E. histolytica* respectively. Click here for file

Additional file 2 **Figure S2.** Orphan C/D box snoRNAs and putative antisense element in mRNAs: Two C/D orphan snoRNAs with possible antisense element (upstream to D' box and/or D box) showed complementary base paring with mRNAs of the indicated genes in *E. histolytica.* Click here for file

Additional file 3 **Figure S3.** Predicted secondary structure of *E. histolytica* snoRNA. Secondary structure of H/ACA box snoRNA (A) and C/D box snoRNA (B) drawn using VARNA visualization tool. Antisense elements are represented by bases colored in green and location of conserved boxes is indicated. Click here for file

Additional file 4 **Table S1.** Oligonucleotides used in this study. Click here for file

Additional file 5 **Figure S4.** Genomic distribution of predicted snoRNAs in *E. histolytica.* Pie chart representing localization of predicted snoRNAs in *E. histolytica* genome*. * Click here for file

Additional file 6 **Figure S5.** H/ACA snoRNAs guiding two sites with single guide sequence: Predicted pseudouridylation guide duplexes between snoRNA and rRNA are shown. The convention followed by [44] has been adopted. snoRNA sequences in a 5’ to 3’ orientation are shown in upper strands, whereas rRNA sequence in 3’ to 5’ orientation are shown in lower strands. The conserved motifs are in bold text. Click here for file

Additional file 7 **Figure S6.** C/D box snoRNAs with predicted antisense element and target RNAs. C/D box snoRNA with two antisense stretch sequence present upstream to D’ and D box (A). Single antisense stretch guiding two different target RNAs (B i-ii). Single antisense stretch guiding different sites in single target RNAs (C i-v). snoRNA sequences in a 3’ to 5’ orientation are shown in lower strand, whereas rRNA sequence in 5’ to 3’ orientation are shown in upper strand. The conserved motifs are in bold text. Click here for file
