# Supplementary figures and images for: Computational prediction and validation of C/D, H/ACA and Eh_U3 snoRNAs of Entamoeba histolytica
Source: BMC Genomics. 2012 Aug 14;13:390. doi: 10.1186/1471-2164-13-390 (PMC3542256; doi:10.1186/1471-2164-13-390)

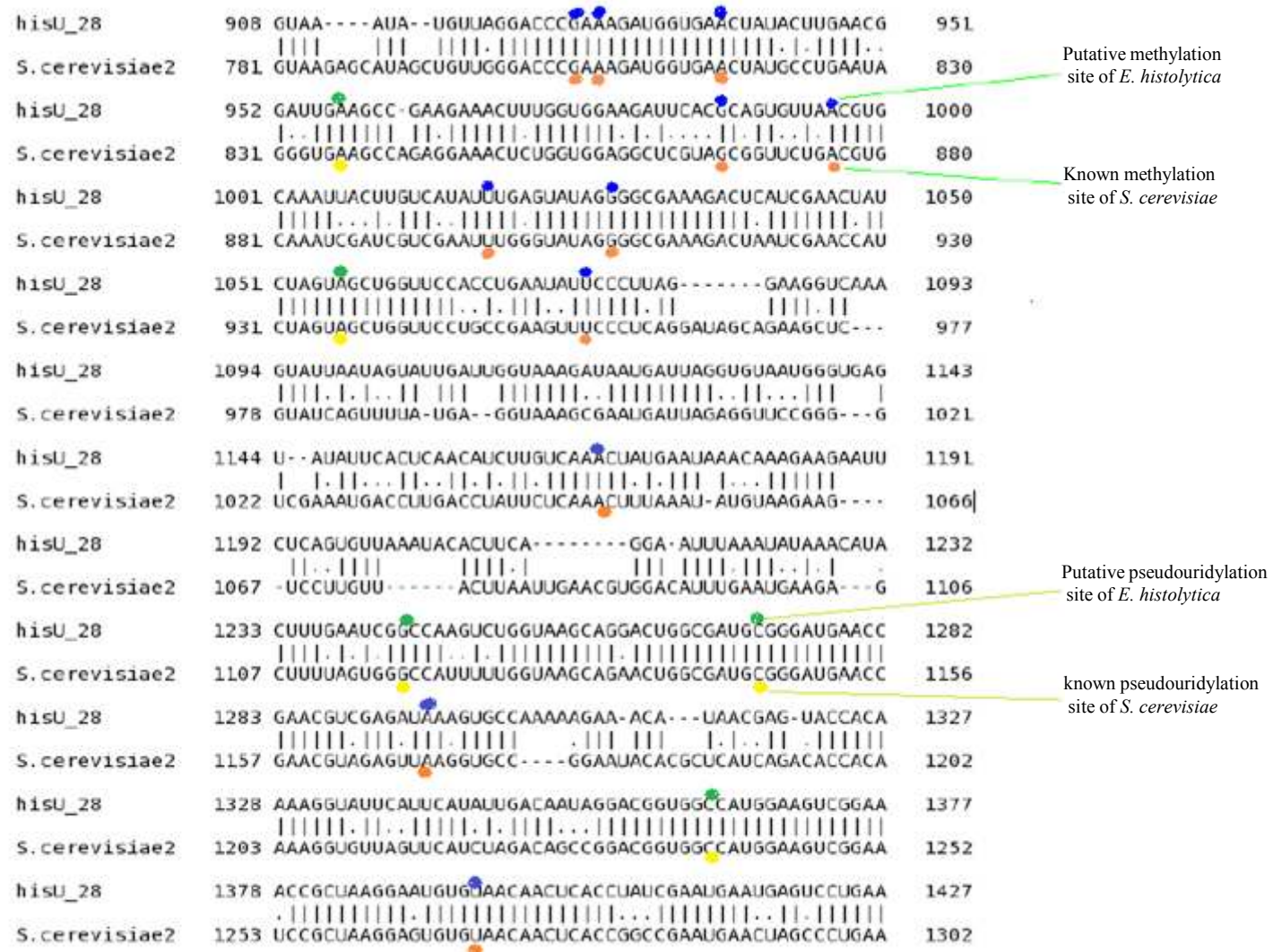

Figure S1

Supplement: Additional file 1 — Figure S1. Global alignment of lsu rRNA of S. cerevisiae and E. histolytica to predict the putative modification sites in E. histolytica. Red and yellow dots are already known methylation and pseudouridylation sites of S. cerevisiae respectively. Blue and green dots are the putative methylation and pseudouridylation sites of E. histolytica respectively. [file 1471-2164-13-390-S1.pdf]

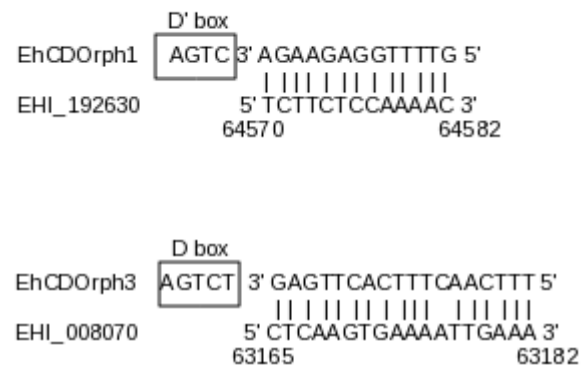

**Figure S2**

Supplement: Additional file 2 — Figure S2. Orphan C/D box snoRNAs and putative antisense element in mRNAs: Two C/D orphan snoRNAs with possible antisense element (upstream to D' box and/or D box) showed complementary base paring with mRNAs of the indicated genes in E. histolytica. [file 1471-2164-13-390-S2.pdf]

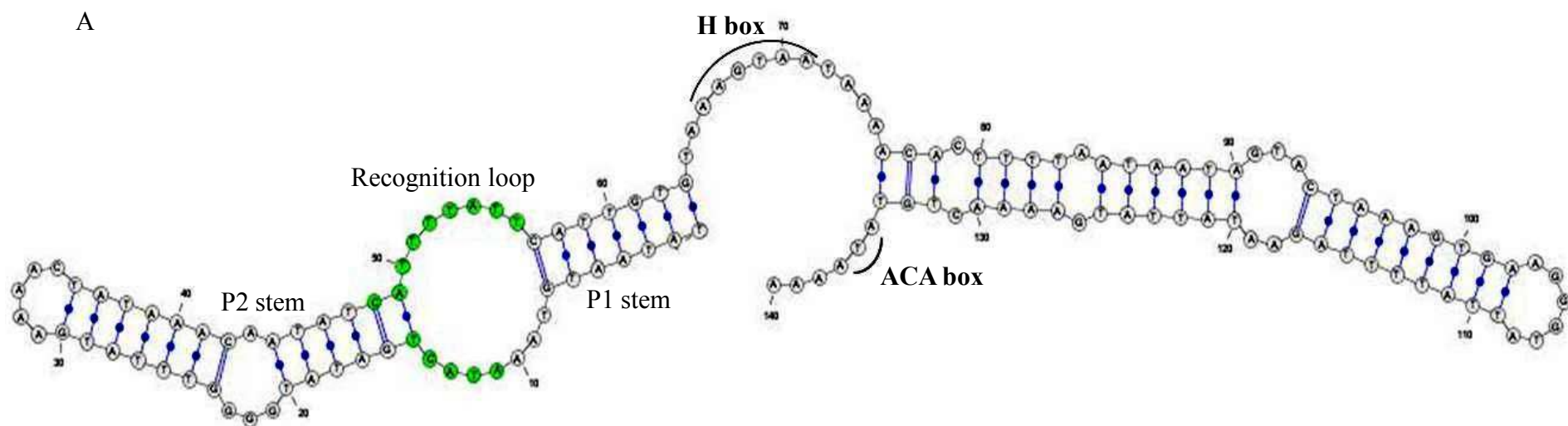

ACA-Eh-5.8S80a

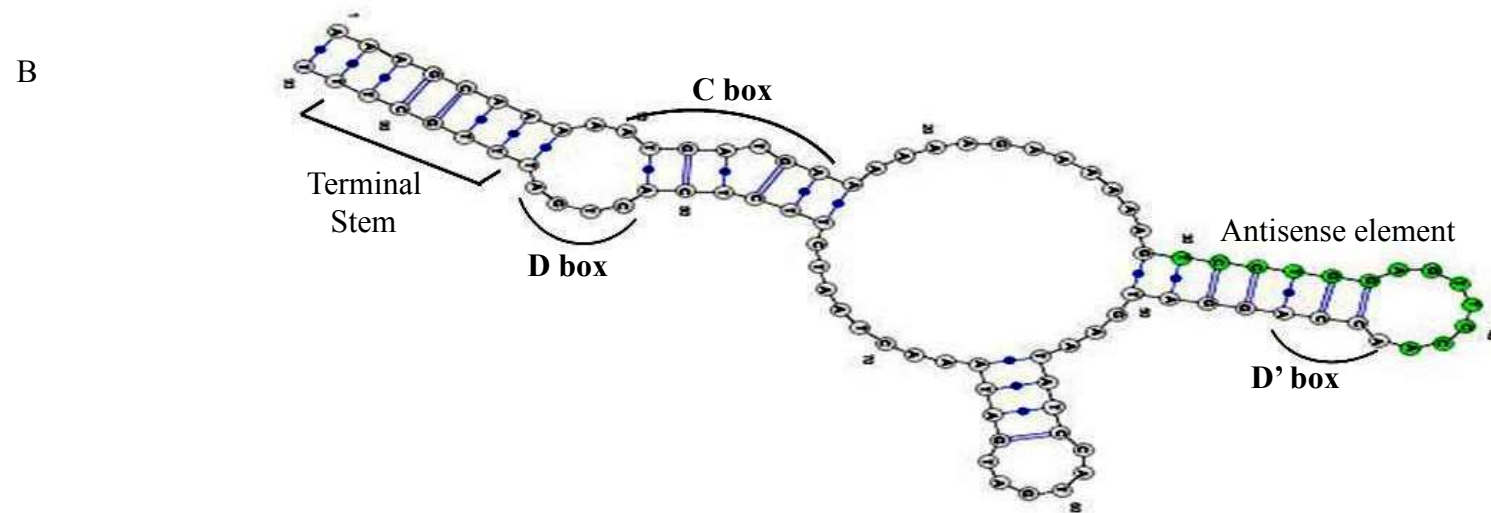

Me-Eh-SSU-A1183

Figure S3

Supplement: Additional file 3 — Figure S3. Predicted secondary structure of E. histolytica snoRNA. Secondary structure of H/ACA box snoRNA (A) and C/D box snoRNA (B) drawn using VARNA visualization tool. Antisense elements are represented by bases colored in green and location of conserved boxes is indicated. [file 1471-2164-13-390-S3.pdf]

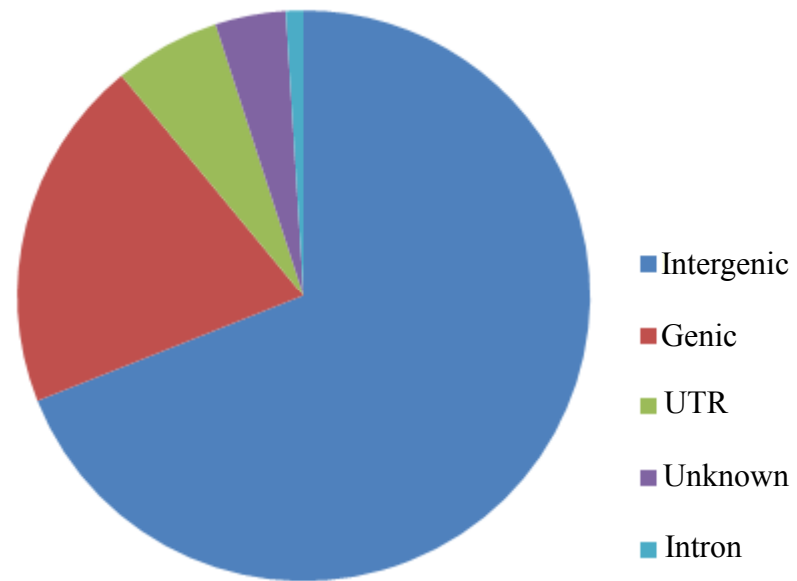

**Figure S4**

Supplement: Additional file 5 — Figure S4. Genomic distribution of predicted snoRNAs in E. histolytica. Pie chart representing localization of predicted snoRNAs in E. histolytica genome. [file 1471-2164-13-390-S5.pdf]

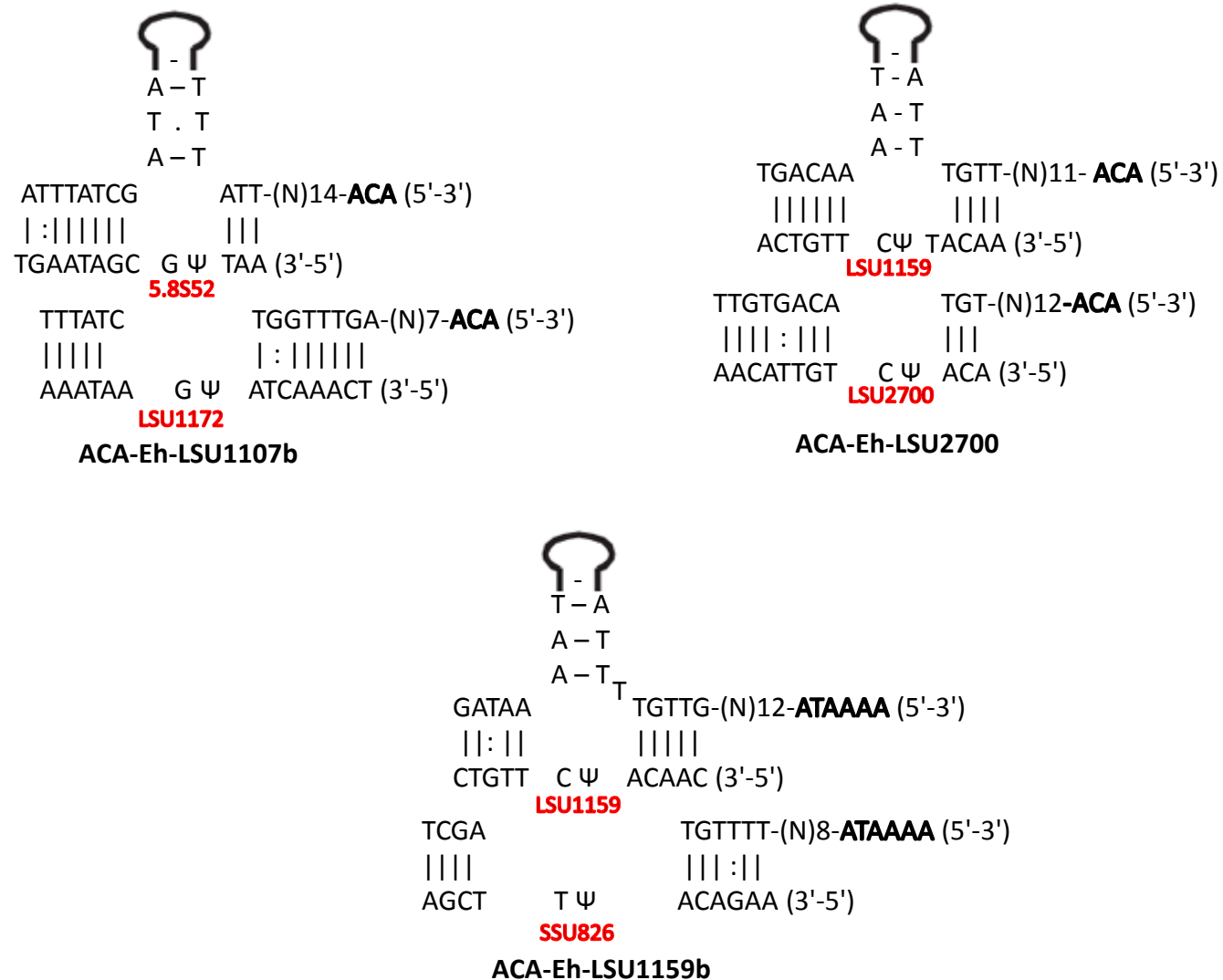

Figure S5

Supplement: Additional file 6 — Figure S5. H/ACA snoRNAs guiding two sites with single guide sequence: Predicted pseudouridylation guide duplexes between snoRNA and rRNA are shown. The convention followed by [44] has been adopted. snoRNA sequences in a 5’ to 3’ orientation are shown in upper strands, whereas rRNA sequence in 3’ to 5’ orientation are shown in lower strands. The conserved motifs are in bold text. [file 1471-2164-13-390-S6.pdf]

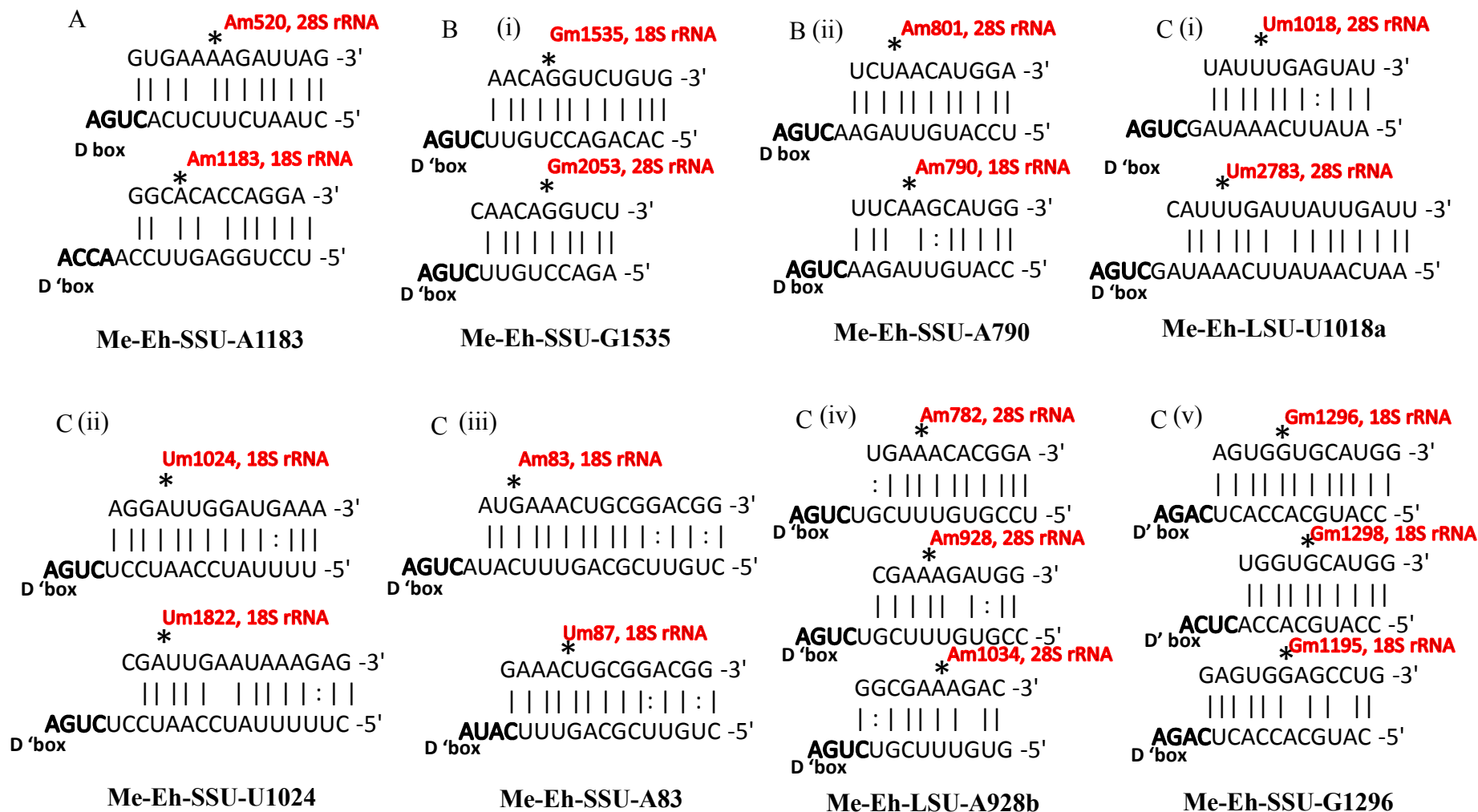

Figure S6

Supplement: Additional file 7 — Figure S6. C/D box snoRNAs with predicted antisense element and target RNAs. C/D box snoRNA with two antisense stretch sequence present upstream to D’ and D box (A). Single antisense stretch guiding two different target RNAs (B i-ii). Single antisense stretch guiding different sites in single target RNAs (C i-v). snoRNA sequences in a 3’ to 5’ orientation are shown in lower strand, whereas rRNA sequence in 5’ to 3’ orientation are shown in upper strand. The conserved motifs are in bold text. [file 1471-2164-13-390-S7.pdf]
